# Identification and validation of cuproptosis-related genes in acetaminophen-induced liver injury using bioinformatics analysis and machine learning

**DOI:** 10.3389/fimmu.2024.1371446

**Published:** 2024-06-27

**Authors:** Zhenya Guo, Jiaping Liu, Guozhi Liang, Haifeng Liang, Mingbei Zhong, Stephen Tomlinson, Songqing He, Guoqing Ouyang, Guandou Yuan

**Affiliations:** ^1^ Division of Hepatobiliary Surgery, The First Affiliated Hospital of Guangxi Medical University, Nanning, Guangxi, China; ^2^ Key Laboratory of Early Prevention and Treatment for Regional High Frequency Tumor (Guangxi Medical University), Ministry of Education, Nanning, Guangxi, China; ^3^ Guangxi Key Laboratory of Immunology and Metabolism for Liver Diseases (Guangxi Medical University), Nanning, Guangxi, China; ^4^ Department of Microbiology and Immunology, Medical University of South Carolina, Charleston, SC, United States

**Keywords:** acetaminophen-induced liver injury, cuproptosis, mitochondria, immune infiltration, machine learning

## Abstract

**Background:**

Acetaminophen (APAP) is commonly used as an antipyretic analgesic. However, acetaminophen overdose may contribute to liver injury and even liver failure. Acetaminophen-induced liver injury (AILI) is closely related to mitochondrial oxidative stress and dysfunction, which play critical roles in cuproptosis. Here, we explored the potential role of cuproptosis-related genes (CRGs) in AILI.

**Methods:**

The gene expression profiles were obtained from the Gene Expression Omnibus database. The differential expression of CRGs was determined between the AILI and control samples. Protein protein interaction, correlation, and functional enrichment analyses were performed. Machine learning was used to identify hub genes. Immune infiltration was evaluated. The AILI mouse model was established by intraperitoneal injection of APAP solution. Quantitative real-time PCR and western blotting were used to validate hub gene expression in the AILI mouse model. The copper content in the mouse liver samples and AML12 cells were quantified using a colorimetric assay kit. Ammonium tetrathiomolybdate (ATTM), was administered to mouse models and AML12 cells in order to investigate the effects of copper chelator on AILI.

**Results:**

The analysis identified 7,809 differentially expressed genes, 4,245 of which were downregulated and 3,564 of which were upregulated. Four optimal feature genes (OFGs; SDHB, PDHA1, NDUFB2, and NDUFB6) were identified through the intersection of two machine learning algorithms. Further nomogram, decision curve, and calibration curve analyses confirmed the diagnostic predictive efficacy of the four OFGs. Enrichment analysis indicated that the OFGs were involved in multiple pathways, such as IL-17 pathway and chemokine signaling pathway, that are related to AILI progression. Immune infiltration analysis revealed that macrophages were more abundant in AILI than in control samples, whereas eosinophils and endothelial cells were less abundant. Subsequently, the AILI mouse model was successfully established, and histopathological analysis using hematoxylin–eosin staining along with liver function tests revealed a significant induction of liver injury in the APAP group. Consistent with expectations, both mRNA and protein levels of the four OFGs exhibited a substantial decrease. The administration of ATTAM effectively mitigates copper elevation induced by APAP in both mouse model and AML12 cells. However, systemic administration of ATTM did not significantly alleviate AILI in the mouse model.

**Conclusion:**

This study first revealed the potential role of CRGs in the pathological process of AILI and offered novel insights into its underlying pathogenesis.

## 1 Introduction

Acetaminophen (APAP) is a widely used antipyretic and analgesic drug in the clinic. Unlike traditional nonsteroidal anti-inflammatory drugs, APAP does not irritate the stomach or intestinal lining. However, APAP has a relatively narrow window of safety. APAP overdose is an important factor that contributes to liver injury and even liver failure (1). At therapeutic doses, most APAP is metabolized into nontoxic glucuronosylated or sulfated metabolites in the liver, and only approximately 5%–9% of APAP is oxidized by cytochrome P450 enzymes into the toxic N-acetyl-p-benzoquinone imine (NAPQI), which is efficiently scavenged by glutathione (GSH) (2). When APAP overdose occurs, excessive NAPQI depletes GSH and binds to the cysteine residues of essential proteins, forming APAP protein adducts. In mitochondria, excessive NAPQI combines with ATP synthase, GSH synthase and respiratory chain enzymes contributing to mitochondrial oxidative stress and dysfunction (2, 3). Mitochondrial oxidative stress and dysfunction are central to APAP-induced liver injury (AILI).

AILI contributes to extensive hepatocyte death, and based on the overwhelming experimental and clinical evidence, the mechanism of APAP-induced cell death should be referred to as programmed necrosis (4). Various forms of programmed cell death such as necroptosis, apoptosis, pyroptosis, and ferroptosis have been reported to potentially participate in APAP-induced cell death (5–8). Cuproptosis, a novel form of programmed cell death, was first described by Tsvetkov et al. at 2022. Tsvetkov et al. first reported that an excessive abundance of copper could trigger a cell death mechanism different from those associated with oxidative stress, such as apoptosis, ferroptosis and necroptosis (9). In cuproptosis, ferrodoxin-1 reduces cupric ions to cuprous ions, which bind to the enzymes involved in regulation of tricarboxylic acid (TCA) cycle, resulting in the excessive aggregation and the loss of Fe-S cluster proteins. Finally, mitochondrial proteotoxic stress occurs and triggers cuproptosis. Interestingly, GSH also plays a protective role in cuproptosis, similar to APAP, functioning as a thiol-containing copper chelator that inhibits this cell death process (9). Copper is mainly stored in the liver and is an essential cofactor for diverse biological processes. Aberrant copper concentrations are involved in many liver diseases (10, 11). In addition, copper plays an important role in the immune response (12). Since the mechanisms of both AILI and cuproptosis depend on mitochondrial dysfunction and are related to GSH, we were intrigued by whether the newly emerging concept of cuproptosis could also contribute to AILI.

Here, we analyzed the expression of cuproptosis-related genes (CRGs) and immune characteristics in 10 AILI model mice and 10 control mice. Machine learning algorithms were used to explore the optimal feature genes (OFGs). The predictive model was validated using a nomogram, decision curve analysis (DCA), and calibration curve analysis. Finally, the relationship between the OFGs and immune infiltration was investigated.

## 2 Materials and methods

### 2.1 Data collection and processing

**Figure 1:**
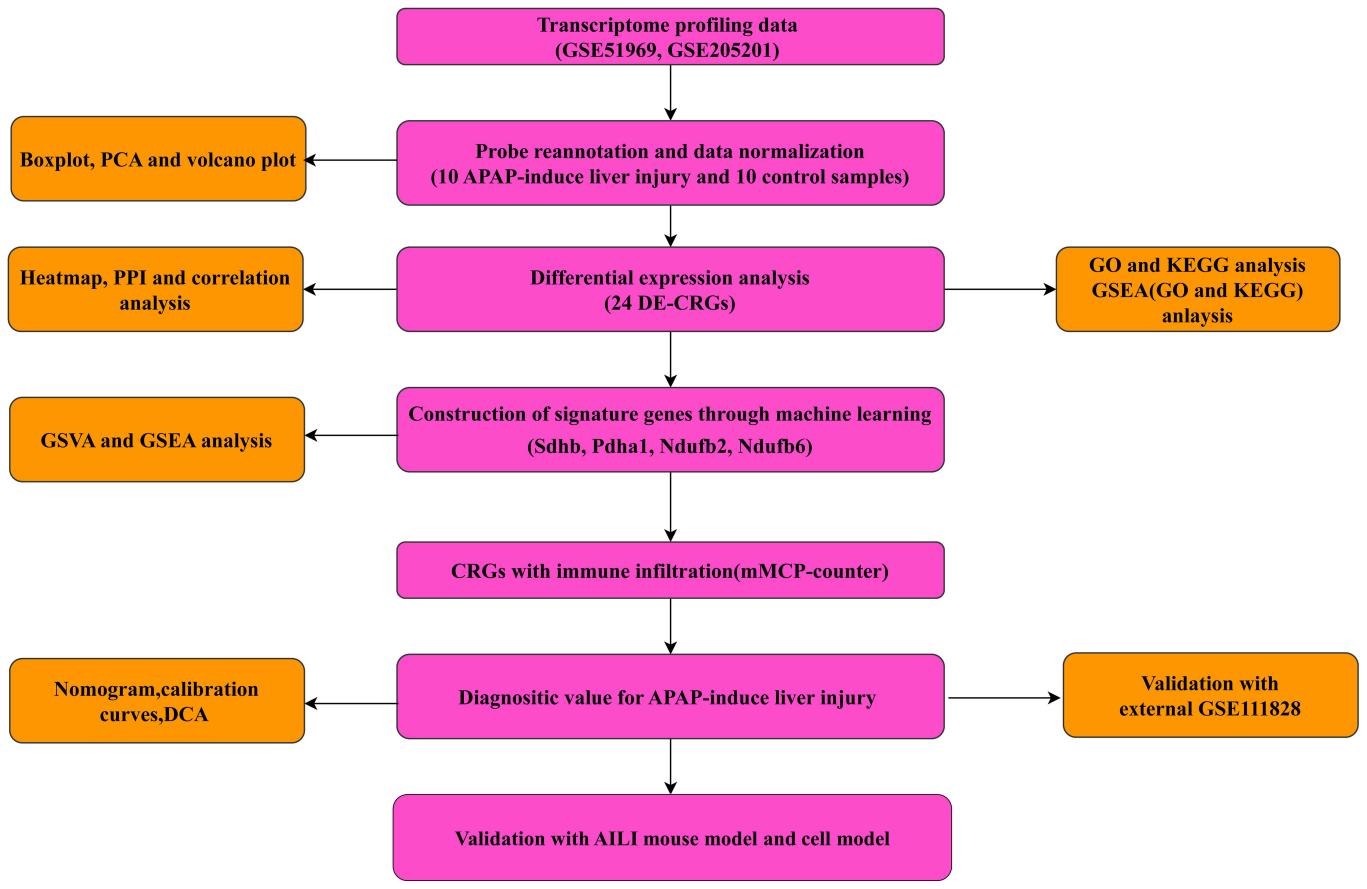
Flowchart of the present study.

The transcriptome profiling data of the AILI and control samples, including the GSE51969 (GPL17226 platform), GSE205201 (GPL29970 platform), and GSE111828 (GPL19057 platform) datasets, were downloaded and selected from the Gene Expression Omnibus database. The GSE51969 and GSE205201 datasets were used to explore the hub genes, and each contained five AILI and five control samples. The GSE111828 dataset was used for validation and included four AILI and four control samples. After merging the GSE51969 and GSE205201 data, the merged datasets contained 10 AILI and 10 control samples. Thirty-five CRGs were retrieved from previous literature (ATP7B, CDKN2A, DLD, DPYD, FDX1, GLRX5, GLS, ISCA2, LIPT1, MTF1, NDUFA1, NDUFA8, NDUFB10, NDUFB2, NDUFB6, NDUFC1, NDUFC2, NDUFV2, PDHA1, PLAT, POLD1, PPAT, SLC31A1, SDHB, TIMMDC1, DLAT, GCSH, DBT, DLST, LIAS, LIPM, LIPA, LIPT2, PDHB, ACO2, NLRP3, and NFE2L2) (11, 13, 14). The flowchart of this study is presented in **Figure 1**.

### 2.2 Differential gene expression analysis of CRGs

The R “limma” package and Wilcoxon signed-rank test was used to explore DEGs between the AILI and control groups. The “ggpubr” R package was used to construct a boxplot of DEGs from 35 CRGs. The results were visualized as volcano and heatmap plots using the “ggplot2” and “pheatmap” R packages. The “VennDiagram” R package was used to present the intersection of DEGs with CRGs. The intersecting genes were defined as DEG-CRGs for subsequent analysis. The genes exhibiting a P value<0.05 and |log_2_FC|>1 was designated as differentially expressed.

### 2.3 Correlation analysis and PPI network construction

A heatmap of 24 DEG-CRGs was generated using the R package “heatmap.” A correlation analysis was performed based on Pearson’s correlation analysis between the DEG-CRGs, and a protein–protein interaction (PPI) network of 24 DEG-CRGs was constructed using the Search Tool for the Retrieval of Interacting Genes/Proteins (STRING) database (https://string-db.org). A medium confidence interval of 0.4 was used for the PPI analysis.

### 2.4 Enrichment analysis of DEG-CRGs

Gene Ontology (GO) and Kyoto Encyclopedia of Genes and Genomes (KEGG) pathway analyses of the 24 DEG-CRGs were performed using the “clusterProfiler” R package and visualized using the R “enrichplot” package. A significant enrichment threshold was set at p < 0.05. Gene set enrichment analysis (GSEA) was also performed to investigate the hub genes’ potential function further (adjust P value<0.05, |log_2_FC|>1). The reference KEGG gene set was selected from the Molecular Signatures Database. Gene set variation analysis (GSVA) was performed to illustrate the differentially enriched gene sets between the high- and low-expression subtypes using the “GSVA” R package. The R “limma” package was used to discover the differentially expressed pathways by comparing GSVA scores between the low- and high-expression subtypes. A p value < 0.05 was considered to indicate significance.

### 2.5 Construction and evaluation of the CRG diagnostic model

The GSE51969 and GSE205201 datasets were merged and used as the training set, while the GSE111828 dataset was used as the validation dataset for the machine learning model. The random forest (RF) algorithm and least absolute shrinkage and selection operator (LASSO) regression were used to screen the OFGs related to AILI prognosis. LASSO regression was performed with the “glmnet” R package (15, 16). The minimum lambda value was set as the optimal value for building the model. The RF model was used to determine the optimal number of variables using various independent decision trees (17), and the RF model was generated using the “randomForest” R package with the “ntree” set at 500. The intersection was used to screen the OFGs derived from the RF and LASSO algorithms. A nomogram model was constructed to predict the occurrence of AILI using the R “rms” package. Each OCG contributes a score, and the “total score” represents the sum of the scores of the OFGs. Calibration curve and decision curve analyses were performed to assess the predictive efficiency of the nomogram model. In addition, clinical impact and decision curves were generated to evaluate the clinical utility of the models. The diagnostic value of the OFGs was evaluated through receiver operating characteristic (ROC) curves generated by calculating the area under the ROC curve (AUC). The R package “pROC” was used to perform the ROC curve analysis (18).

### 2.6 Evaluating immune infiltration

The Microenvironment Cell Populations-counter (mMCP-counter) method (19) was used to estimate the fractions of 13 types of immune cells in each sample from the merged dataset, and to evaluate the correlation between the OFGs and immune cells. The results were visualized with box-plots and heatmaps. A p value < 0.05 was considered to indicate significance.

### 2.7 AILI mouse model construction

Eight-week-old male C57BL/6J mice were purchased from GemPharmatech (Jiangshu, China). All animal experiments were performed according to the Animal Care and Use Committee of Guangxi Medical University. The mice were fasted for 12 h before dosing and then injected intraperitoneally with either normal saline (NS) or 300 mg/kg APAP solution. The mice were sacrificed 24 h after injection. Fresh liver and blood samples were collected immediately for the subsequent analyses.

### 2.8 Cell culture and treatment

AML12 cells were obtained from Procell (CL-0602, Procell, China) and cultured in specialized medium (CM-0602, Procell, China) in a humidified atmosphere of 5% CO2 at 37°C. The establishment of APAP-induced cellular damage was achieved by treating AML12 cells with a 10 mM APAP solution for 24 hours (20).

### 2.9 Cell viability assay

Cellular viability was analyzed using the cell counting kit-8 (C6005M, UElandy, China) according to the manufacturer’s instructions.

### 2.10 Validation of the OFGs in the AILI mouse model

Formalin-fixed, paraffin-embedded liver tissue sections were stained with hematoxylin–eosin (HE). Percent necrosis areas were estimated in five randomly selected high power fields (200×) per sample, with the mean representing the degree of liver necrosis in each sample. Serum alanine aminotransferase (ALT) and aspartate aminotransferase (AST) levels were measured with an autoanalyzer (catalyst one, IDEXX, USA). Total RNA was extracted from liver tissues using TRIzol reagent (15596026, Invitrogen) according to the manufacturer’s instructions. cDNA was synthesized using a PrimeScript RT reagent kit (RR036A, TaKaRa), followed by quantitative PCR with iTaq Universal SYBR Green Supermix (172–5124, Bio-Rad) on a real-time PCR system (CFX 96 Touch, Bio-Rad). The expression of each OFG was normalized to that of GAPDH in the same sample. The primers used are listed in **Supplementary Table 1**. Western blotting (WB) was performed to measure the protein expression of the OFGs as previously described (21), with antibodies against NDUFB2 (17614–1-AP, Proteintech), NDUFB6 (16037–1-AP, Proteintech), PDHA1 (18068–1-AP, Proteintech), and SDHB (10620–1-AP, Proteintech). β-actin (81115–1-RR, Proteintech) was used as an internal control.

### 2.11 Detection of the hepatic copper and the administration of ammonium tetrathiomolybdate

The copper content in the mouse liver samples were quantified using a colorimetric assay kit (E-BC-K300-M; Elabscience) according to the instruction. To investigate the effects of copper chelator on AILI, ATTM (HY-W076067, MedChemExpress) was administered to mouse models. Briefly, the mice received oral gavage of 10 mg/kg (22, 23) of ATTM once daily for three consecutive days and were subsequently utilized to establish an AILI mouse model following the aforementioned protocol.

The copper content in the AML12 cells was quantified using a colorimetric assay kit (E-BC-K775-M; Elabscience) according to the instruction. Before the detection, AML12 cells were treated with ATTM (10 µM) (24, 25), APAP (10 mM), or a combination of ATTM and APAP for 24 hours.

### 2.12 Statistical analyses

The statistical and data analyses were performed utilizing R software (version 4.2.1). Normally distributed continuous variables are expressed as the means ± standard deviations (SDs) and were compared between two groups by two-tailed Student’s t test, while the Wilcoxon rank-sum test was used for nonnormally distributed variables. A two-tailed P value of less than 0.05 was considered to indicate significance.

## 3 Results

### 3.1 Identification of cuproptosis-related genes involved in AILI

**Figure 2:**
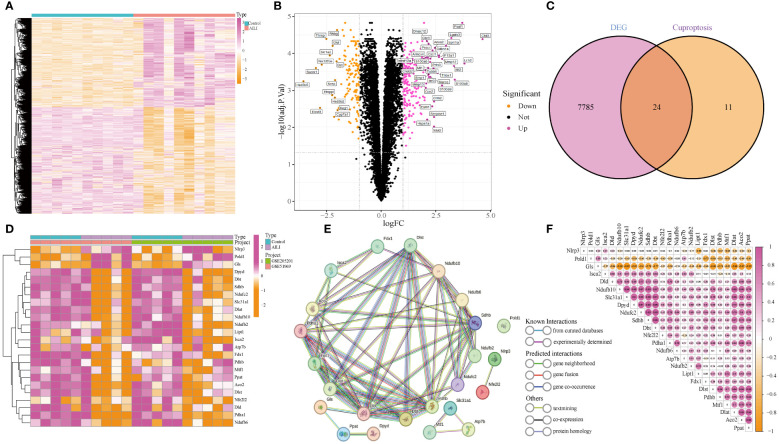
Identification of DEG-CRGs between the AILI and control samples. **(A, B)** Heatmap and volcano plots of the DEGs. **(C)** Venn diagram showing the intersection of genes between DEGs and CRGs. **(D)** Heatmap of the 24 DEG-CRGs. **(E)** PPI analysis of the 24 DEG-CRGs. **(F)** Correlation analysis of the 24 DEG-CRGs. Red and yellow represent positive and negative correlations respectively. AILI, acetaminophen induced liver injury; DEGs, differentially expressed genes; CRGs, cuproptosis-related genes; DEG-CRGs, intersection of DEGs with CRGs; PPI, protein–protein interaction.

Two datasets (GSE51969 and GSE205201), including 10 AILI and 10 control samples, were merged and batch-normalized (**Supplementary Figures 1A–D**). A total of 7,809 DEGs were identified, 4,245 of which were downregulated and 3,564 of which were upregulated. A heatmap and the volcano plots of the DEGs are shown in **Figures 2A, B**. Overlapping the 7,809 DEGs with 35 CRGs revealed 24 DEG-CRGs (NLRP3, POLD1, GLS, DPYD, DBT, SDHB, NDUFC2, SLC31A1, DLAT, NDUFB10, NDUFB2, LIPT1, ISCA2, ATP7B, FDX1, PDHB, MTF1, PPAT, ACO2, DLST, NFE2L2, DLD, PDHA1, and NDUFB6; **Figure 2C**). The data showed that NLRP3, POLD1, and GLS were upregulated in AILI, while the other DEG-CRGs were downregulated (**Figure 2D**). To investigate the potential crosstalk between these DEG-CRGs, we performed PPI analyses using STRING (**Figure 2E**). The correlations between the 24 DEG-CRGs are depicted in **Figure 2F**. Nlrp3, Pold1, and Gls were negatively associated with most of the other DEG-CRGs. The interrelationship between the other 21 DEG-CRGs was positive (**Figure 2F**).

### 3.2 Enrichment analysis of the DEG-CRGs

GO and KEGG enrichment analyses were performed on the 24 DEG-CRGs to determine their biological functions and pathways using the “ClusterProfiler” package. The results are presented in **Supplementary Figure 2**. The data suggested that the 24 DEG-CRGs were significantly involved in lipoic acid metabolism, the TCA cycle, 2-oxocarboxylic acid metabolism, carbon metabolism, and other metabolic pathways, such as oxidative phosphorylation, glycolysis, and pyruvate metabolism.

### 3.3 Construction of diagnostic marker genes for AILI

**Figure 3:**
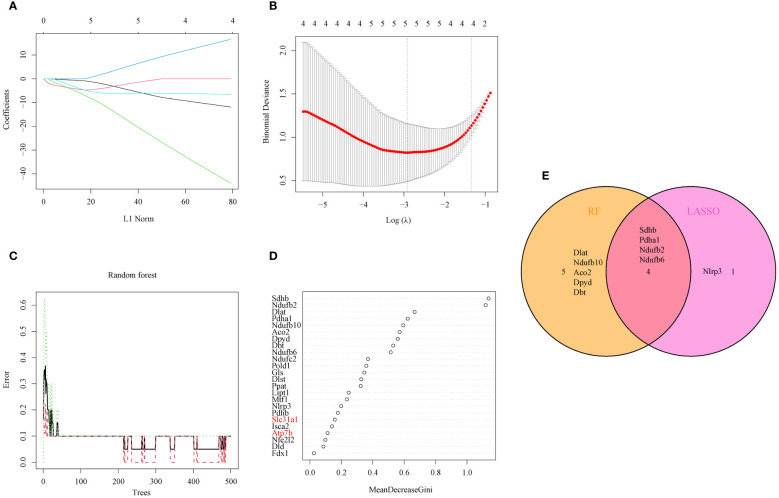
Identification of OFGs for AILI through machine learning. **(A)** Ten-fold cross-validation of tuning parameter selection in the LASSO model. Each curve represents one gene. **(B)** LASSO coefficient analysis. The dotted vertical line is drawn at the optimal lambda. **(C)** Relationships between the number of random forest trees and the number of errors. **(D)** RF algorithm for OFG selection (genes highlighted in red, copper transporter). **(E)** Venn diagram showing the overlap of OFGs between LASSO and random forest analyses. AILI, acetaminophen induced liver injury; OFGs, optimal feature genes; RF, random forest; LASSO, least absolute shrinkage and selection operator.

The LASSO logistic regression and RF algorithms were used to identify OFGs from the 24 DEG-CRGs to determine critical markers with high diagnostic value. First, five OFGs were obtained via LASSO logistic regression (**Figures 3A, B**). Then, the support vector machine (SVM) model and RF model were explained using the “DALEX” package. The data indicated that the RF model had a lower residual weight (**Supplementary Figures 3A, B**). Moreover, the RF model had a greater AUC (SVM, AUC = 0.99; RF, AUC = 1.00; **Supplementary Figure 3C**). Since the RF model had better performance, it was used to filter out nine OFGs (**Figures 3C, D**). Finally, the LASSO and RF OFGs were intersected, and four OFGs were identified (SDHB, PDHA1, NDUFB2, and NDUFB6) for further analysis (**Figure 3E**).

### 3.4 Validation of the OFGs

**Figure 4:**
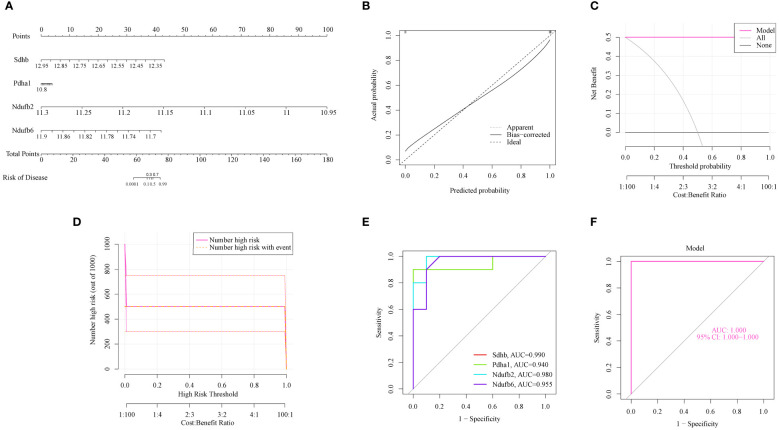
The predictive efficiency of the OFGs. **(A)** Nomogram of the four OFGs. **(B)** Calibration curve showing the diagnostic ability of the nomogram model. **(C)** DCA illustrating the predictive efficiency of the nomogram models. **(D)** Clinical impact curve showing the greater diagnostic ability of the nomogram model. **(E)** ROC results for the four OFGs. **(F)** A logistic regression model was used to determine the AUC of AILI. AILI, acetaminophen induced liver injury; ROC, receiver operating characteristic; AUC, area under the ROC curves; DCA, decision curve analysis; OFGs, optimal feature genes.

To further assess the predictive efficiency, a nomogram model was constructed using the “rms” package using SDHB, PDHA1, NDUFB2, and NDUFB6 (**Figure 4A**). Each OCG was assigned a score, and AILI risk was predicted using the cumulative score. The calibration curve and DCA were applied for assessing the predictive efficiency of the nomogram model. The calibration curves suggested a relative link between the predicted and actual probabilities (**Figure 4B**). DCA indicated that the nomogram model had significantly greater net benefits than the individual OCG, suggesting a high level of accuracy and providing a foundation for physician decision-making (**Figure 4C**). The clinical impact curve also indicated that the nomogram model has a relatively high diagnostic ability (**Figure 4D**). The ROC curves showed that the individual OCGs had high diagnostic value (SDHB, AUC = 0.99; PDHA1, AUC = 0.94; NDUFB2, AUC = 0.98; NDUFB6, AUC = 0.955; **Figure 4E**). Moreover, the four OFGs demonstrated a significantly greater diagnostic value (AUC = 1, **Figure 4F**). These results indicate that the diagnosis model is efficacious in distinguishing AILI from normal individuals.

**Figure 5:**
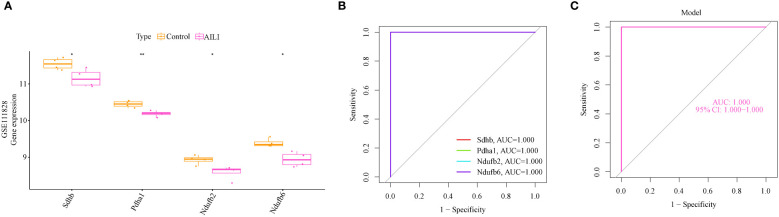
Validation of the OFGs in the GSE111828 dataset. **(A)** Boxplots indicating that the four OFGs were significantly altered between the AILI and control samples **(B)** ROC curves of the four OFGs in the GSE111828 dataset. **(C)** ROC curve analysis of the four-OFG-based model in the GSE111828 dataset. P values are shown as * p < 0.05 and ** p < 0.01. OFGs, optimal feature genes; ROC, receiver operating characteristic.

The gene expression and ROC curves of the four OFGs were validated using the GSE111828 dataset for further verification. The results showed that the expression of the four OFGs was significantly downregulated (**Figure 5A**). Additionally, the ROC curve analysis demonstrated that these OFGs, individually and together, demonstrated powerful predictive abilities (AUC = 1, **Figures 5B, C**).

### 3.5 GSVA and GSEA

GSVA and GSEA of the KEGG enrichment analysis were performed to detect the differentially active pathways between the low- and high-expression subtypes according to the expression level of the four OFGs (**Supplementary Figure 4**). Interestingly, the data indicated that all four OFGs were related to cytokine cytokine receptor interaction and the IL-17 signaling pathway. GSVA and GSEA of the OFGs based on the GO enrichment also were performed (**Supplementary Figure 5**), and the results showed that the OFGs were involved with immune system process and inflammatory response. These findings implied that the OFGs may play a crucial role in the regulation of immune inflammatory response in AILI.

### 3.6 Immune infiltration analysis

**Figure 6:**
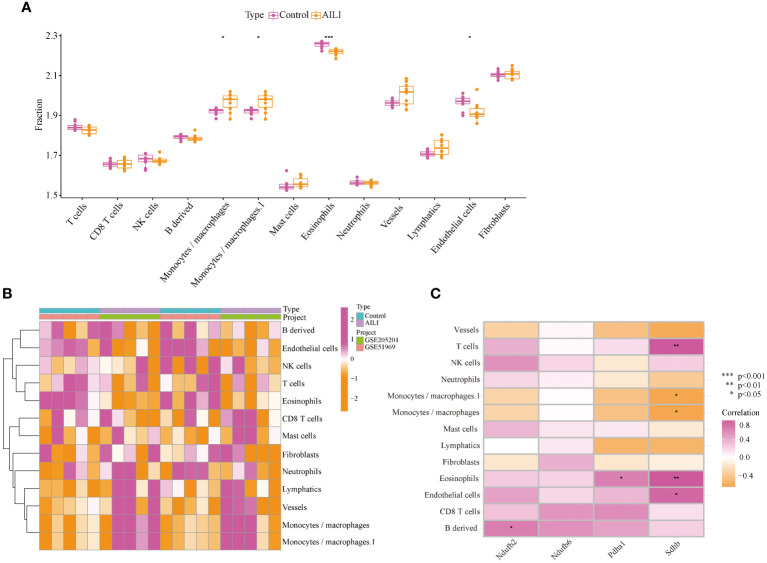
Immune infiltration analysis of the AILI and control samples. **(A)** Box plots showing the differences in immune infiltration between the AILI and control samples. **(B)** Heatmap of immune cells. **(C)** Correlation analysis between the OFGs and immune cells. P values are shown as * p < 0.05, ** p < 0.01, and *** p < 0.001. AILI, acetaminophen induced liver injury; OFGs, optimal feature genes.

Studies have shown that various immune cells, such as macrophages, neutrophils, and natural killer cells, play pivotal roles in AILI. Notably, CRGs also play a regulatory role in the immune response (26, 27). Our GSVA and GSEA results further substantiate the pivotal role of OFGs in regulating immune inflammatory response in AILI. Therefore, we evaluated the immune microenvironment through the mMCP-counter method. The data indicated that macrophages were more abundant in the AILI group than in the control group, whereas eosinophils and endothelial cells were less abundant (**Figure 6A**). A heatmap of the immune cells is presented in **Figure 6B**. Correlation analysis revealed positive correlations between NDUFB2 and B-cell-derived cells, and between PDHA1 and eosinophils. SDHB appears more extensively involved in regulating the immune microenvironment. The analysis revealed that SDHB was positively correlated with T cells, eosinophils, and endothelial cells, and negatively correlated with macrophages (**Figure 6C**). These results suggest that modifications in the immune microenvironment may contribute to AILI development.

### 3.7 OFG expression in AILI

**Figure 7:**
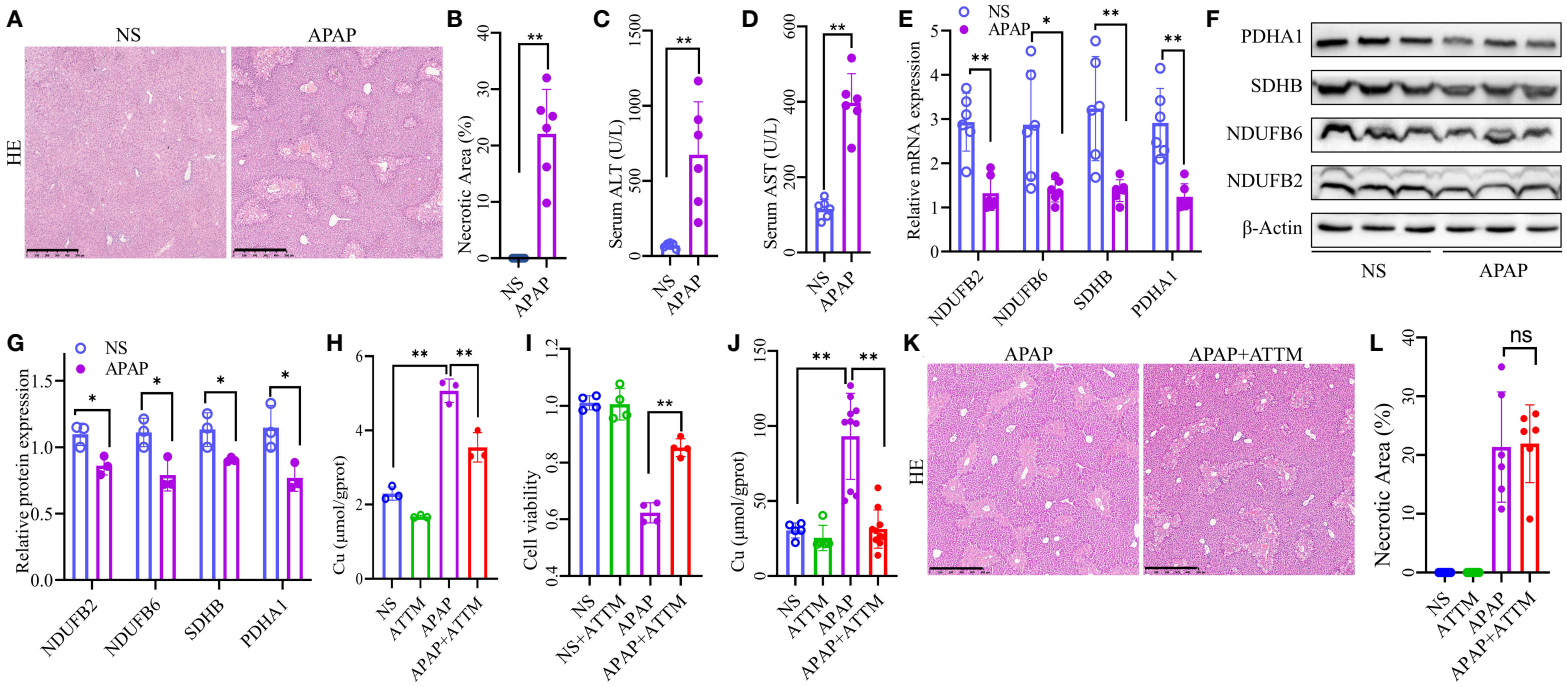
Altered expression of the OFGs and effects of ATTM on AILI. **(A, B)** HE staining to assess liver necrosis (Scale bar, 500 µm; n = 6 per group). **(C, D)** Serum concentrations of ALT and AST (n = 6 per group). **(E)** Quantitative PCR analysis of NDUFB2, NDUFB6, PDHA1, and SDHB (n = 6 per group). **(F, G)** Western blotting and densitometry analysis of NDUFB2, NDUFB6, PDHA1, and SDHB (n = 3 per group). **(H)** Copper levels of AML12 cells (n = 3 for each group). **(I)** Cell viability of AML12 cells (n = 4 for each group). **(J)** Hepatic copper levels of AILI mouse model (n = 5 for NS and ATTM group, n = 10 for APAP and APAP+ATTM group). **(K, L)** HE staining to assess liver necrosis (Scale bar, 500 µm; n = 6 per group). The results are expressed as the mean ± SD. P values are shown as * p < 0.05 and ** p < 0.01; ns, not significant. AILI, acetaminophen induced liver injury; OFGs, optimal feature genes; ALT, alanine aminotransferase; AST, aspartate aminotransferase; HE, hematoxylin-eosin staining; PCR, polymerase chain reaction; ATTM, ammonium tetrathiomolybdate.

We established an AILI mouse model and further verified the expression of the four OFGs. HE demonstrated massive hepatic necrosis in the livers of the APAP-treated mice (**Figures 7A, B**). Similarly, liver function tests showed that the serum ALT and AST levels were significantly higher in the APAP group than in the NS group (**Figures 7C, D**). We evaluated the expression of the four OFGs at both the mRNA and protein levels. As expected, the expression of SDHB, PDHA1, NDUFB2, and NDUFB6 in the APAP group was significantly decreased at both mRNA and protein levels (**Figures 7E–G**).

### 3.8 Detection of hepatic copper levels and the effects of ATTM on AILI

The accumulation of intracellular copper is a pivotal characteristic of cuproptosis. Therefore, we sought to investigate whether the administration of copper chelator could effectively ameliorate AILI by reducing copper levels. The results of *in vitro* cell experiments revealed a significant increase in copper levels within the APAP group compared to the NS group, whereas ATTM exhibited a remarkable ability to effectively mitigate the elevated copper levels (**Figure 7H**). Correspondingly, the subsequent experiments demonstrated that ATTM could significantly alleviate APAP-induced inhibition on the cellular viability of AML12 cells (**Figure 7I**). To further validate the effects of ATTM on AILI, we performed additional experiments using AILI mouse model. As expected, the hepatic copper levels were significantly higher in the APAP group than in the NS group, and ATTM could significantly reduce hepatic copper levels in the context of AILI (**Figure 7J**). However, ATTM did not significantly alleviate AILI. In fact, ATTM seemed to have a tendency to worsen AILI (**Figures 7K, L**; **Supplementary Figure 6**).

## 4 Discussion

Drug-induced liver injury (DILI) is a global problem caused by various commonly used drugs. Some of the drugs often implicated in DILI are APAP, aspirin, and cocaine (28). Due to the widespread application of APAP, AILI has become a significant threat to public health. The mechanism underlying AILI is complex and multifactorial and involves liver metabolism, mitochondrial oxidative stress and dysfunction, sterile inflammation, and autophagy. Cuproptosis is a newly discovered type of apoptosis mainly characterized by the aberrant accumulation of cellular copper above a certain threshold, which induces cell toxicity and, eventually, cell death (9). Cuproptosis plays an important role in numerous diseases, such as Wilson’s disease, neurodegenerative diseases, and cancer (10). Notably, mitochondrial oxidative stress and dysfunction are the major cellular events involved in both AILI and cuproptosis. However, whether cuproptosis plays a role in the pathogenesis of AILI has not been well investigated. Our present study aimed to identify potential CRGs that may contribute to AILI.

In this study, 24 DEG-CRGs were identified, and enrichment analysis indicated that the DEG-CRGs were significantly related to aerobic respiration, iron-sulfur cluster binding, the mitochondrial respirasome, the citrate cycle, carbon metabolism, and pyruvate metabolism. Tsvetkov et al. revealed that increased copper accumulation causes the lipoylation and aggregation of enzymes (especially DLAT, which is critical for the formation of the multienzyme pyruvate dehydrogenase complex) involved in the regulation of the mitochondrial TCA cycle (9). The destabilization of Fe-S cluster proteins is another remarkable feature of cuproptosis. Our bioinformatics analysis results are highly consistent with those of previous studies on the mechanism of cuproptosis. Additionally, we used machine learning models to identify OFGs for the diagnosis of AILI according to the expression profiles of 24 DEG-CRGs. A total of four OFGs were identified, namely, SDHB, PDHA1, NDUFB2, and NDUFB6. The predictive efficacy was verified by constructing a nomogram, generating calibration curves, and performing DCA. Theoretically, the four-gene model could be a reliable and robust biomarker for predicting AILI.

SDHB encodes the iron–sulfur protein subunit of succinate dehydrogenase (complex II), which is a key enzyme in the TCA cycle and the electron transport chain. SDHB deficiency increases mitochondrial copper concentrations and contributes to increased oxidative stress (29, 30). These findings are consistent with our bioinformatics analysis results showing that SDHB, a CRG, is significantly downregulated in AILI and may promote oxidative stress in this disease. In addition, the decreased expression of SDHB may increase mitochondrial copper levels in AILI, indicating that cuproptosis may be involved in AILI. PDHA1 encodes the alpha 1 subunit of pyruvate dehydrogenase, which catalyzes the conversion of pyruvate to acetyl-CoA and serves as a significant bridge between glycolysis and the TCA cycle. Our findings demonstrated that the expression level of PDHA1 is decreased in AILI. Theoretically, decreased PDHA1 expression may lead to decoupling of glycolysis from the TCA cycle. Interestingly, a metabolic profiling study on AILI revealed that it is associated with the decoupling of glycolysis from the TCA cycle, the loss of nicotinamide adenine dinucleotide phosphate (NADPH) production, and the suppression of anabolism (31). The authors of the metabolic profiling study proposed that APAP toxicity may be caused by the decoupling of glycolysis from the TCA cycle, lactic acidosis, reduced NADPH production, and subsequent suppression of the anabolic pathways required for rapid growth (31). These implied that PDHA1 and AILI may be related through the metabolic profile remodeling. NDUFB2 and NDUFB6 encode the subunits of the nicotinamide adenine dinucleotide (NADH): ubiquinone oxidoreductase (complex I). Mammalian complex I has NADH dehydrogenase activity and oxidoreductase activity. It has been reported that APAP toxicity occurs with the loss of mitochondrial membrane potential and decreased NADH levels (1). Furthermore, NADPH-dependent APAP-GSH conjugate production was synergistically enhanced by NADH (32). In AILI, both NDUFB2 and NDUFB6 were decreased, which may inhibit the clearance of APAP by GSH.

In this study, we analyzed immune infiltration in AILI samples. The ratio of total to M1 macrophages was significantly higher in the AILI group than in the control group. It has been reported that after APAP overdose, the number of M1 macrophages in the liver of rats increases significantly with increasing M1-related cytokines, such as IFN-γ and TNF-α (33). It is well known that proinflammatory M1 macrophages are involved in various liver diseases, especially in the pathological processes of oxidative stress and sterile inflammatory responses. Thus, the associations between M1 macrophage polarization and damage-associated molecular patterns (DAMPs) and autophagy might contribute to AILI pathogenesis (33). The anti-inflammatory M2 macrophages also were observed, but their activation was significantly delayed (33). Bioinformatics analysis revealed that the number of eosinophils was significantly decreased in AILI samples. However, these findings contradict previous findings. Xu et al. reported that both the percentage and the total number of eosinophils were increased in the livers of mice treated with APAP and proposed that eosinophils were recruited into the liver and played a profound protective role (34). This contradiction may be related to differences in pretreatment and sampling times; however, further investigations are needed. Our data indicated that endothelial cells are also significantly decreased in AILI. Another study confirmed that hepatic endothelial cells are an early and direct target for APAP hepatotoxicity (35). It is reasonable to speculate that APAP-induced endothelial cell injury may be responsible for the decrease in endothelial cells. Further correlation analysis revealed a significant correlation between the OFGs and multiple immune cells. SDHB, in particular, appears to be more extensively involved in regulating the immune response. The aberrant expression of SDHB could disrupt the assembly of mature complex II, which is involved in multiple mitochondrial processes, including oxidative phosphorylation, pyruvate metabolism, the citric acid cycle, and phospholipid metabolism (36, 37). It has become increasingly recognized that mitochondrial function is involved in the differentiation and activation of immune cells. For example, proinflammatory macrophages exhibit a break at complex II in the TCA cycle, leading to the accumulation of succinate (38). The accumulation of succinate is further linked to the induction of a proinflammatory phenotype through autocrine stimulation of succinate receptor 1, which activates inflammatory pathways by promoting IL-1β production (39). Studies have also demonstrated that mitochondrial metabolism is necessary for T-cell activation, proliferation, and function. For instance, loss of complex II could inhibit the production of IFN-γ by Th1 cells (40). Briefly, mitochondrial respiratory-related genes such as SDHB may take a part in the pathological course of AILI.

The experimental results suggested a potential involvement of cuproptosis in AILI. Interestingly, although ATTM demonstrates a significant improvement in the cell viability of AML12 cells, it does not exhibit any ameliorative effects on AILI in mouse model. Nonetheless, we consider that this result does not invalidate the potential relationship between cuproptosis and AILI. Copper plays an essential cofactor in innate immunity and metabolism. Previous studies have demonstrated that copper deficiency is an independent risk factor for mortality in patients with advanced liver disease (41). Moreover, another study revealed that although copper levels are elevated in various liver fibrosis conditions, severe copper deficiency induced by tetrathiomolybdate exacerbates liver injury and fibrosis in rats (22). Therefore, it can be speculated that systemic administration of copper chelators may not be a feasible approach to inhibiting cuproptosis for alleviating AILI. Taking a step back, even if the systemic administration of copper chelators could effectively inhibit hepatic cuproptosis, it might still pose potential harm rather than benefit. AILI involves complex and diverse mechanisms beyond cuproptosis alone, thus maintaining a relatively normal physiological environment with appropriate copper levels could potentially offer more benefits for AILI.

## 5 Conclusions

In summary, our present study revealed the relationship between CRGs and immune cells in the pathological process of AILI. Four OFGs were identified using a machine-learning model. Our research provides novel insights into the role of CRGs in AILI and a better understanding of the underlying pathogenesis mechanism of this disease. Nevertheless, our study has several limitations. The datasets in this study were all obtained from an AILI mouse model, and the sample size was small. In addition, the research lacked clinical sample data, which would be more convincing. Finally, we could not explore the regulatory mechanism of OFGs in AILI. Hence, further investigations are needed in the future.

## Data availability statement

The original contributions presented in the study are included in the article/**Supplementary Material**. Further inquiries can be directed to the corresponding authors.

## Ethics statement

The animal study was approved by Animal Care and Use Committee of Guangxi Medical University. The study was conducted in accordance with the local legislation and institutional requirements.

## Author contributions

ZG: Writing – review & editing, Writing – original draft, Software, Funding acquisition, Formal analysis, Data curation. JL: Writing – review & editing, Software, Data curation. GL: Writing – review & editing, Data curation. HL: Writing – review & editing, Data curation. MZ: Writing – review & editing, Data curation. ST: Writing – review & editing. SH: Writing – review & editing, Project administration, Funding acquisition. GO: Writing – original draft, Software, Formal analysis, Data curation. GY: Writing – review & editing, Project administration, Funding acquisition.
