# Supplementary material for: Identification and validation of cuproptosis-related genes in acetaminophen-induced liver injury using bioinformatics analysis and machine learning
Source: Front Immunol. 2024 Jun 27;15:1371446. doi: 10.3389/fimmu.2024.1371446 (PMC11236684; doi:10.3389/fimmu.2024.1371446)

Identification and validation of cuproptosis-related genes in acetaminophen-induced liver injury using bioinformatics analysis and machine learning

Zhenya Guo^1,2,3,#^, Jiaping Liu^1,2,3,#^, Guozhi Liang^1,2,3^, Haifeng Liang^1,2,3^, Mingbei Zhong^1,2,3^, Stephen Tomlinson^4^, Songqing He^1,2,3^*, Guoqing Ouyang^1,2,3^*, [Guandou Yuan](https://pubmed-ncbi-nlm-nih-gov-443--bjmu.jitui.me/?term=Yuan+G&cauthor_id=35069557)^1,2,3^*

^1^Division of Hepatobiliary Surgery, The First Affiliated Hospital of Guangxi Medical University, Nanning, Guangxi 530021, China.

^2^Key Laboratory of Early Prevention and Treatment for Regional High Frequency Tumor (Guangxi Medical University), Ministry of Education, Nanning, Guangxi 530021, China.

^3^Guangxi Key Laboratory of Immunology and Metabolism for Liver Diseases, Nanning, Guangxi 530021, China.

^4^Department of Microbiology and Immunology, Medical University of South Carolina, Charleston, SC, United States.

^#^ These authors have contributed equally to this work

*Correspondence to:

Guandou Yuan, Division of Hepatobiliary Surgery, The First Affiliated Hospital of Guangxi Medical University, NO 6 Shuangyong Road, Nanning 530021, Guangxi, China. E-mail: [dr_yuangd@gxmu.edu.cn](mailto:dr_yuangd@gxmu.edu.cn)

Guoqing Ouyang, Division of Hepatobiliary Surgery, The First Affiliated Hospital of Guangxi Medical University, NO 6 Shuangyong Road, Nanning 530021, Guangxi, China. Email: [Ouyangguoqing@stu.gxmu.edu.cn](mailto:Ouyangguoqing@stu.gxmu.edu.cn).

Songqing He, Division of Hepatobiliary Surgery, The First Affiliated Hospital of Guangxi Medical University, NO 6 Shuangyong Road, Nanning 530021, Guangxi, China. Email: [dr_hesongqing@163.com](mailto:dr_hesongqing@163.com)

Contents

目录

[Supplementary Table 1 3](#_Toc164900107)

[Supplementary Figure 1 4](#_Toc164900108)

[Supplementary Figure 2 5](#_Toc164900109)

[Supplementary Figure 3 6](#_Toc164900110)

[Supplementary Figure 4 7](#_Toc164900111)

[Supplementary Figure 5 11](#_Toc164900112)

[Supplementary Figure 6 15](#_Toc164900113)

# Supplementary Table 1

The primers for GAPDH, SDHB, PDHA1, NDUFB2, and NDUFB6.

| Genes | | Sequences (5’-3’) |
| --- | --- | --- |
| GAPDH | Forward | ACTCCACTCACGGCAAATTC |
|  | Reverse | TCTCCATGGTGGTGAAGACA |
| SDHB | Forward | AATTTGCCATTTACCGATGGGA |
|  | Reverse | AGCATCCAACACCATAGGTCC |
| PDHA1 | Forward | GAAATGTGACCTTCATCGGCT |
|  | Reverse | TGATCCGCCTTTAGCTCCATC |
| NDUFB2 | Forward | CCCCGGTACAGGGAGTTTC |
|  | Reverse | GCCAAAATCGCCAAAGAATCCA |
| NDUFB6 | Forward | TGGAAGAACATGGTCTTTAAGGC |
|  | Reverse | TTCGAGCTAACAATGGTGTATGG |

# Supplementary Figure 1

Two datasets (GSE51969 and GSE205201), including 10 AILI and 10 control samples, were merged and batch-normalized. (A, B) Before batch correlation. (C, D) After batch correlation. AILI, acetaminophen induced hepatic injury.


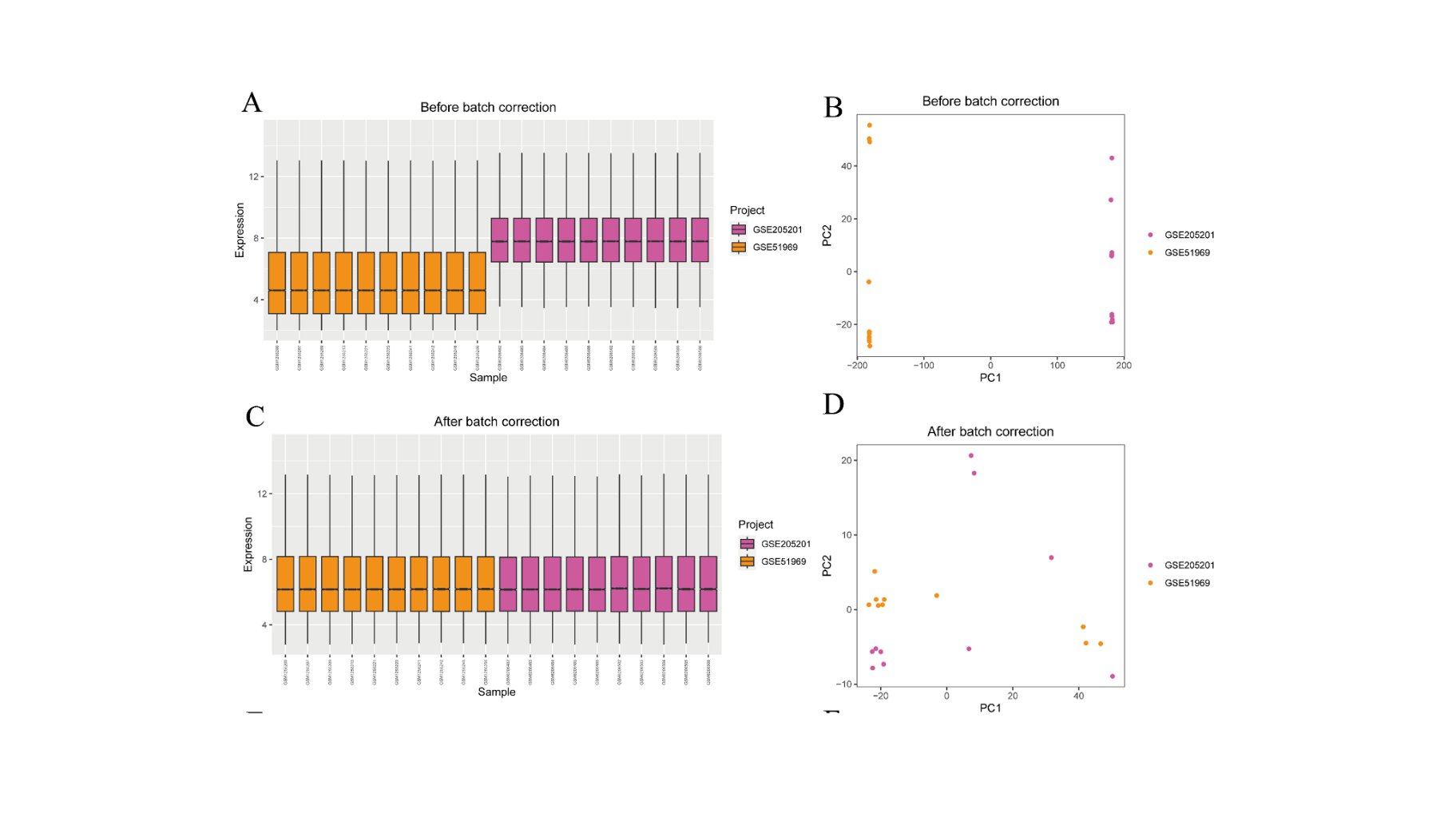


Supplementary Figure 2

Enrichment analyses of the DEG‐CRGs. (A) GO enrichment analysis of the 24 DEG-CRGs. (B) KEGG enrichment analysis of the 24 DEG-CRGs. (C, D) GSEA revealed five critical biological functions and five critical signaling pathways enriched in the control group. (E, F) GSEA revealed six critical biological functions and six critical signaling pathways enriched in AILI group. AILI, acetaminophen induced liver injury; GO; Gene Ontology; KEGG, Kyoto Encyclopedia of Genes and Genomes; GSEA, Gene Set Enrichment Analysis.


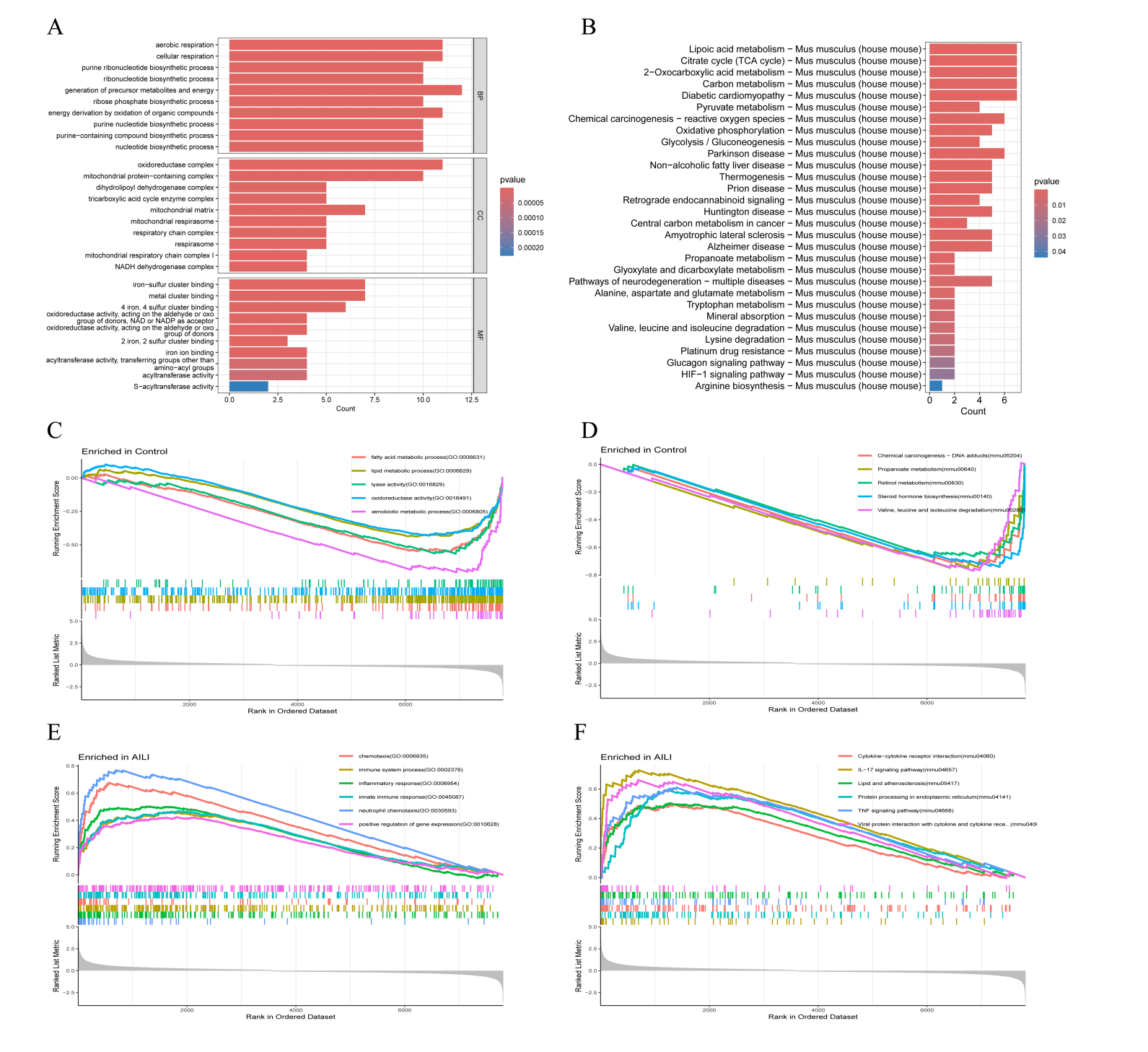


# Supplementary Figure 3

The SVM model and RF model were explained using the “DALEX” package, and the RF model had a better performance. (A) Boxplots showing the residuals of each machine learning model. (B) Cumulative residual distribution of each machine learning model. (C) ROC analysis of machine learning models. SVM, support vector machine; RF, random forest; ROC, receiver operating characteristic.


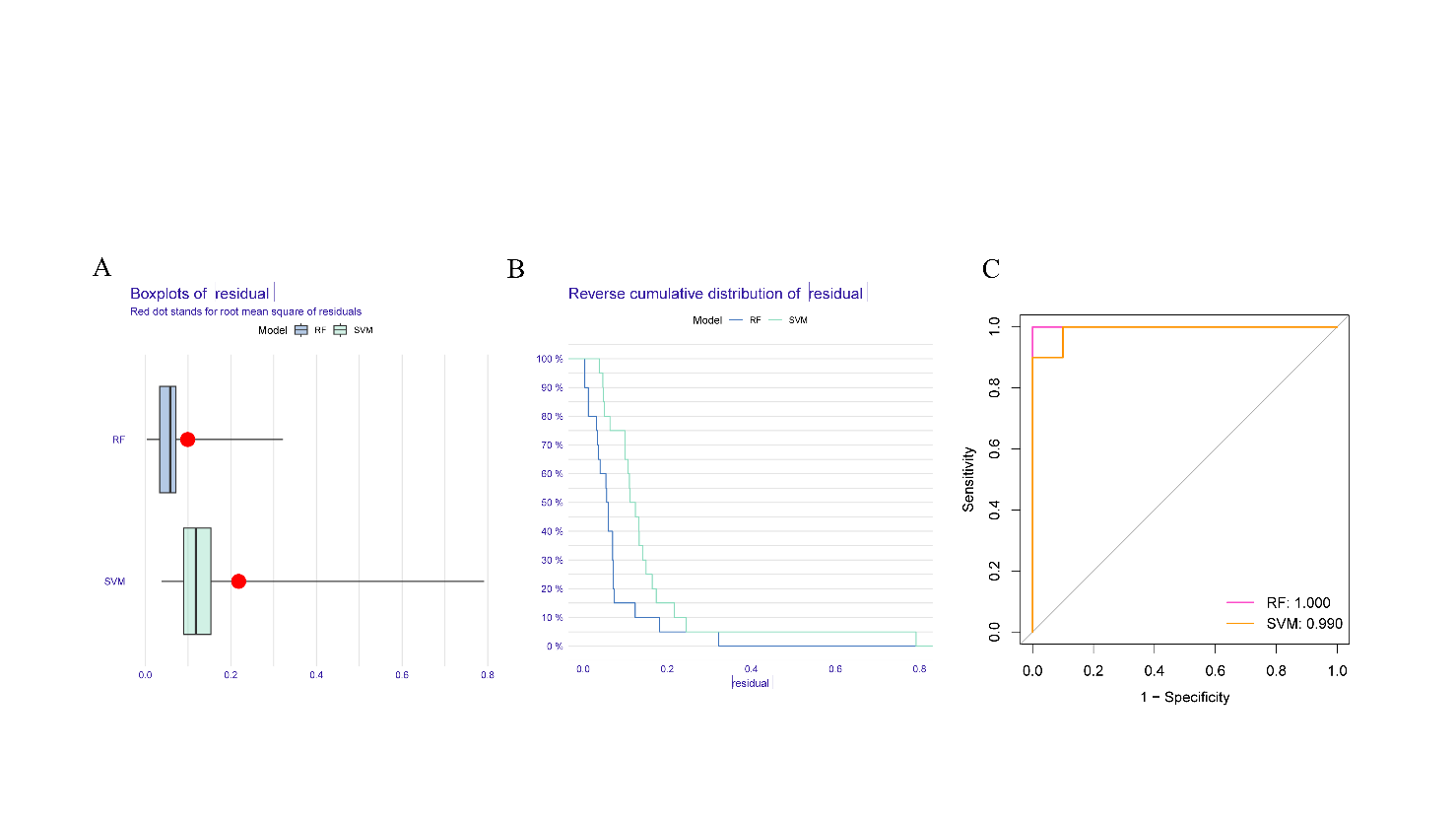


Supplementary Figure 4

GSVA and GSEA of the OFGs based on the KEGG enrichment. KEGG pathway enrichment analysis of (A) NDUFB2, (B) NDUFB6, (C) PDHA1, and (D) SDHB was conducted using the GSVA method. KEGG pathway enrichment analysis of (E) NDUFB2, (F) NDUFB6, (G) PDHA1, and (H) SDHB was conducted using the GSEA method. GSVA, Gene set variation analysis; GSEA, gene set enrichment analysis; OFGs, optimal feature genes; KEGG, Kyoto Encyclopedia of Genes and Genomes.

4-A (NDUFB2)


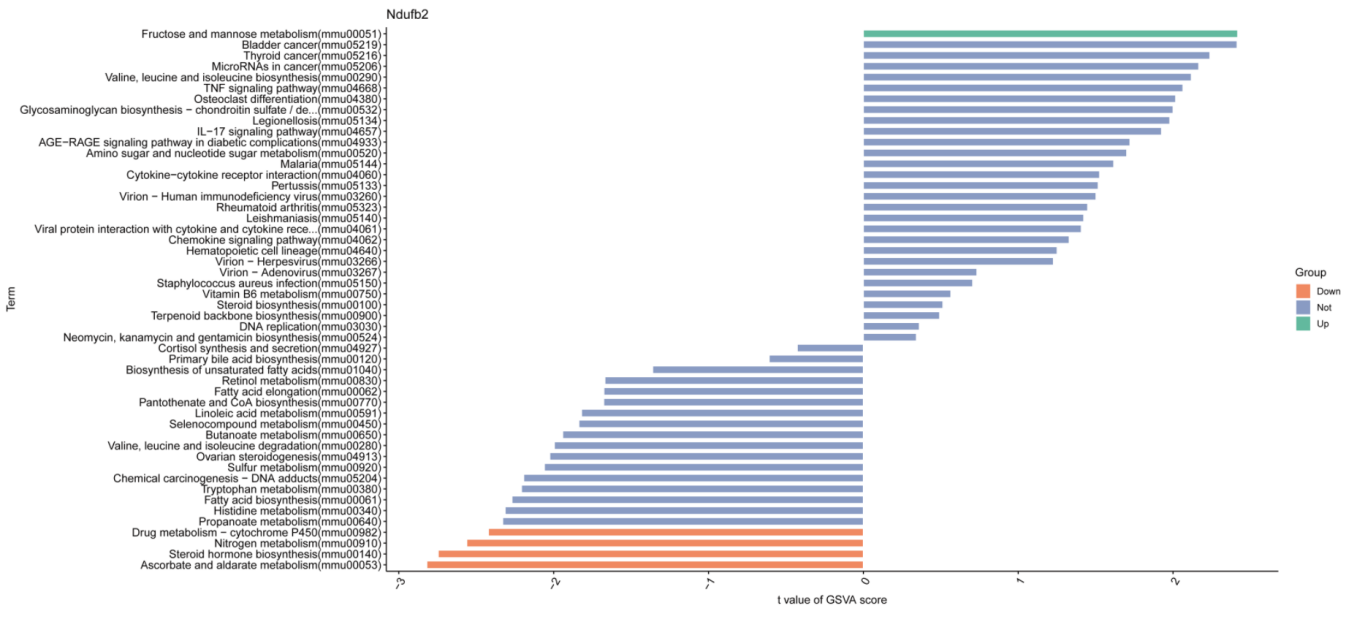


4-B (NDUFB6)


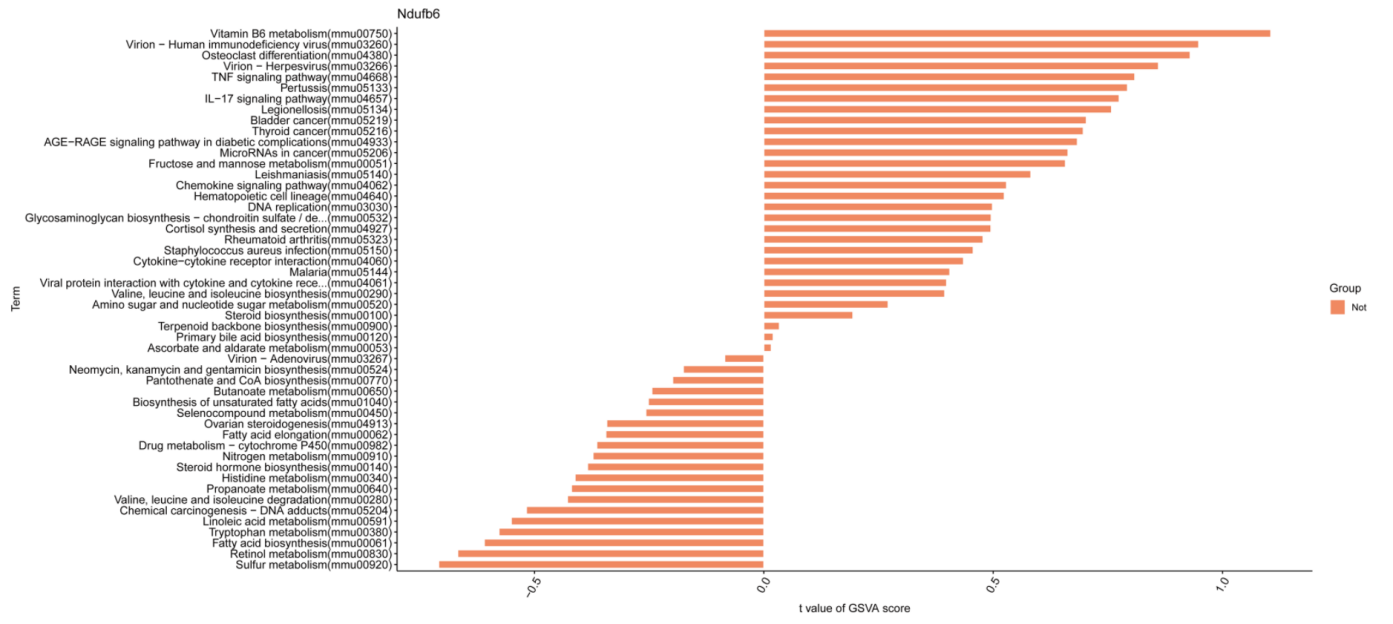


4-C (PDHA1)


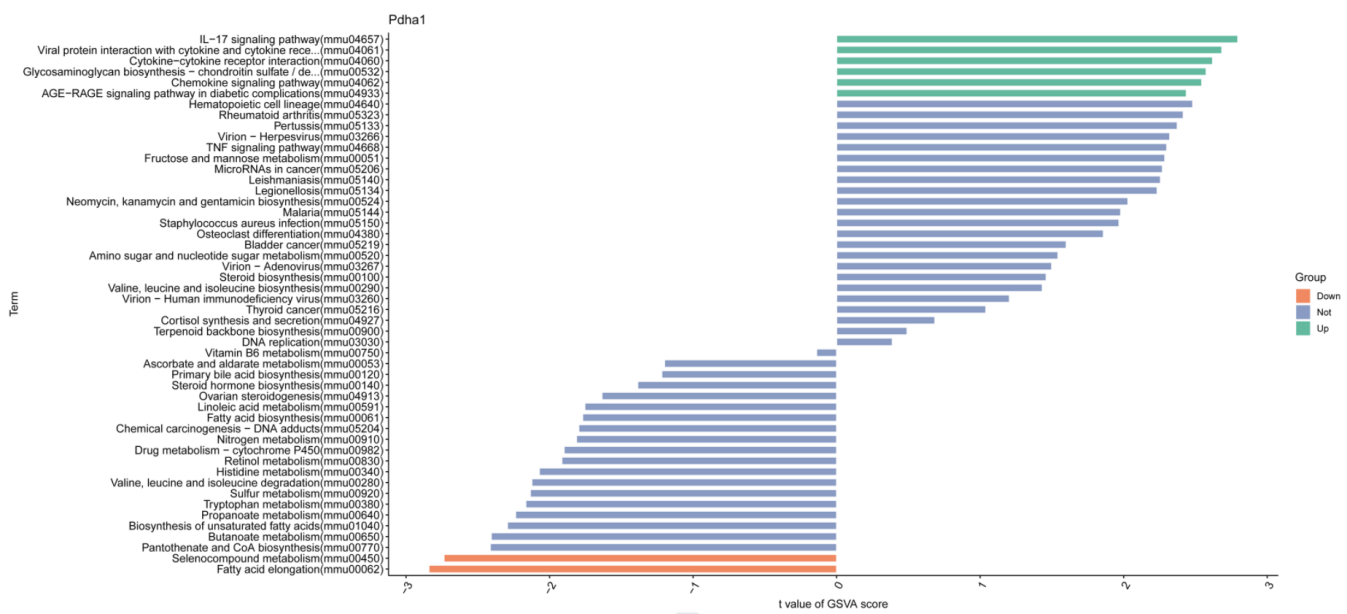


4-D (SDHB)


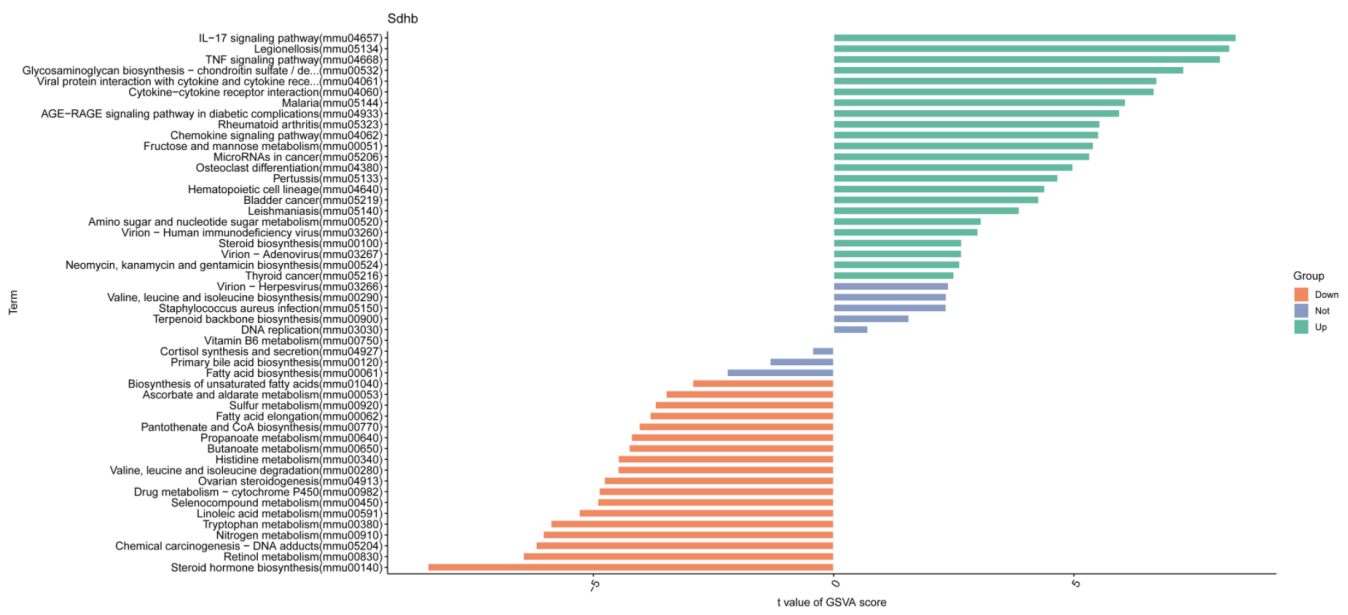


4-E (NDUFB2)


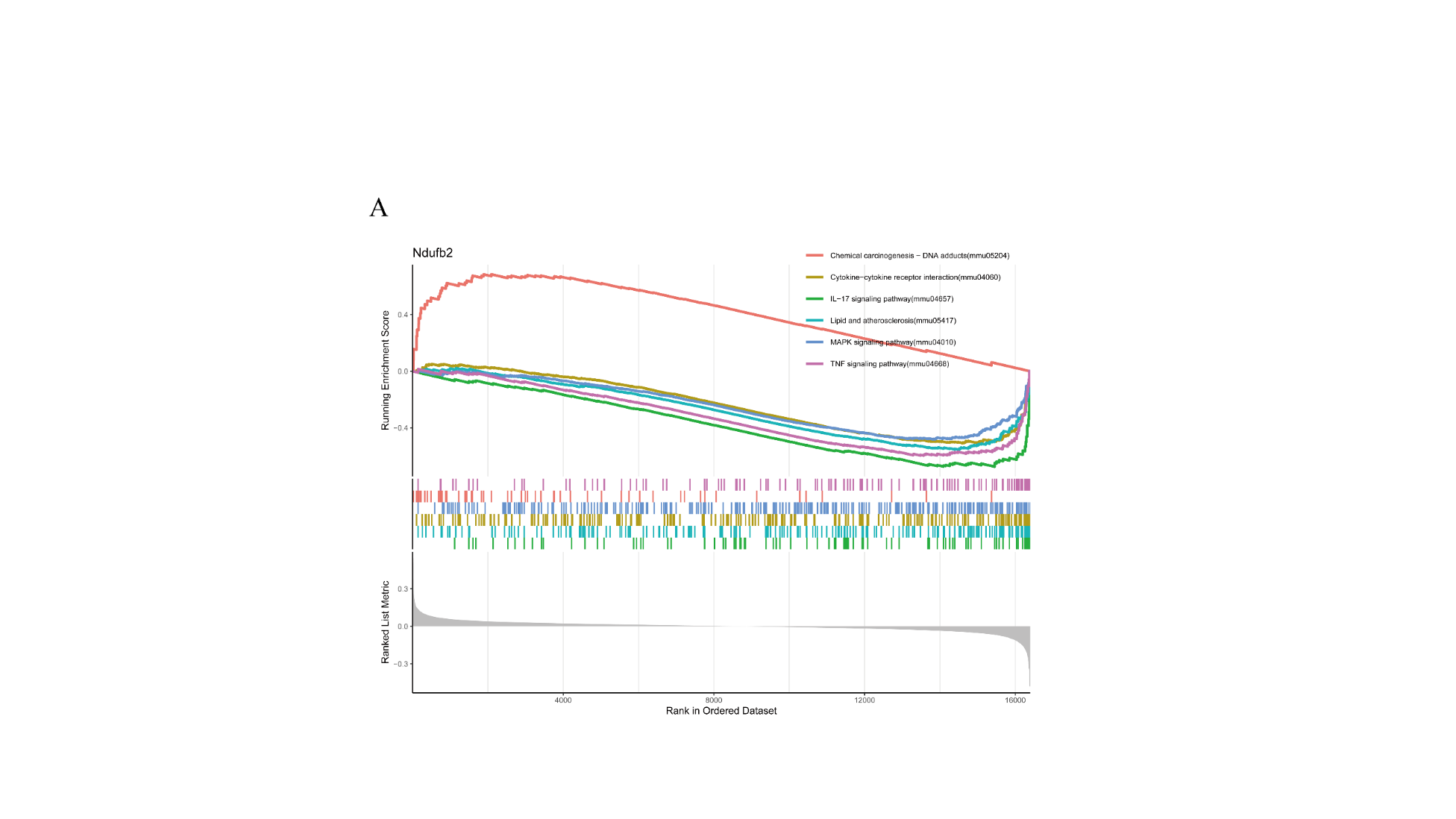


4-F (NDUFB6)


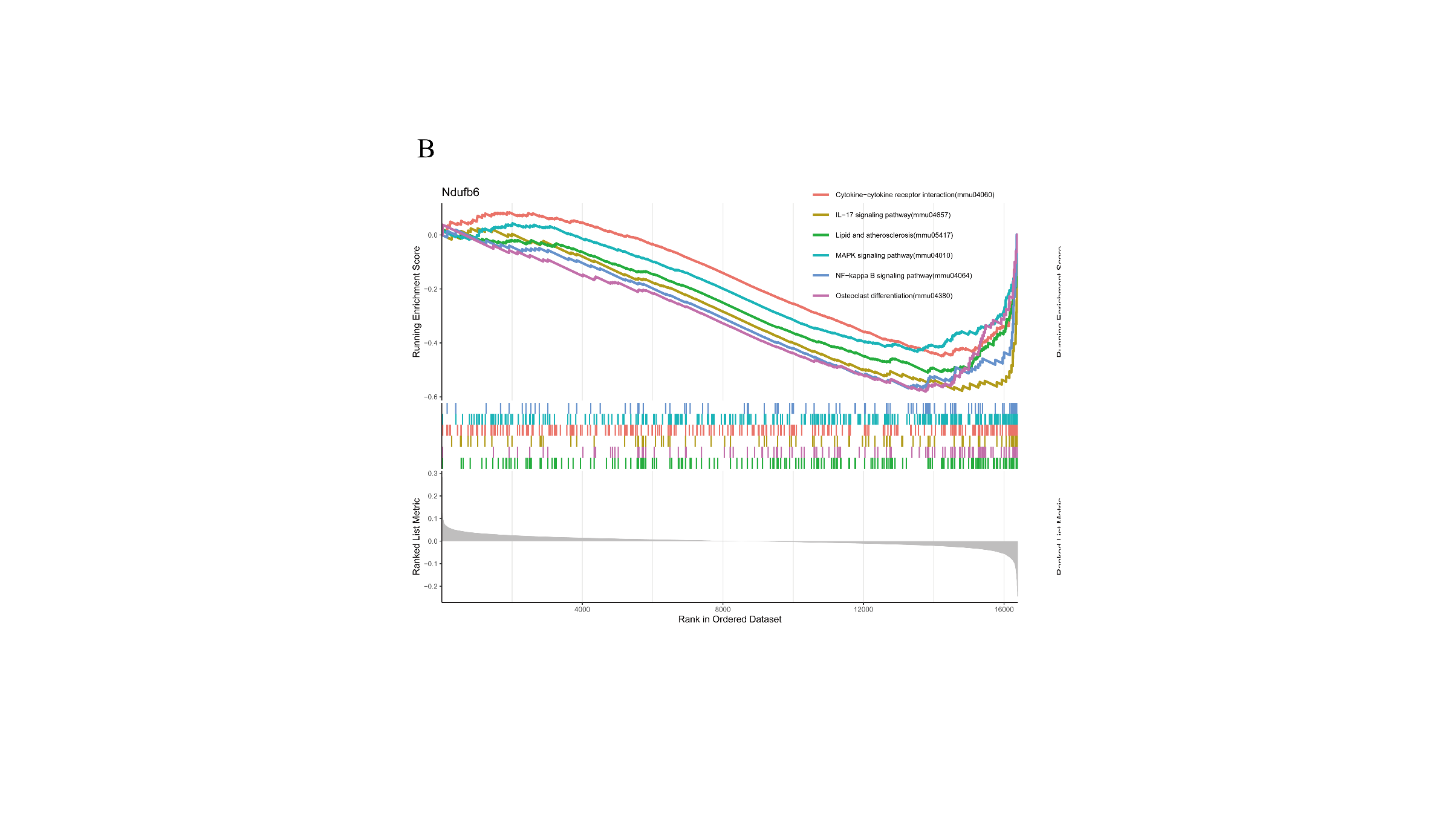


4-G (PDHA1)


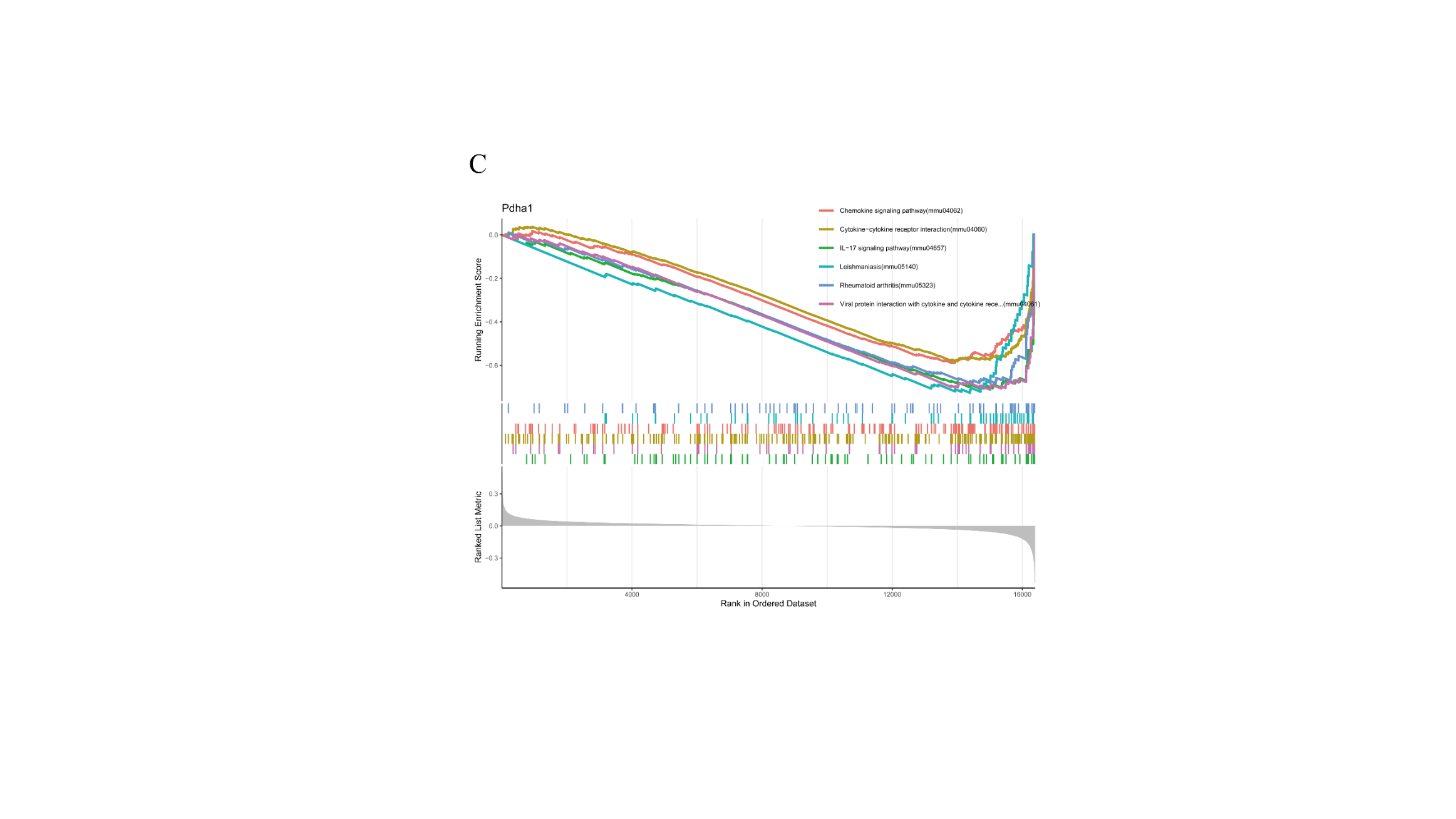


4-H (SDHB)


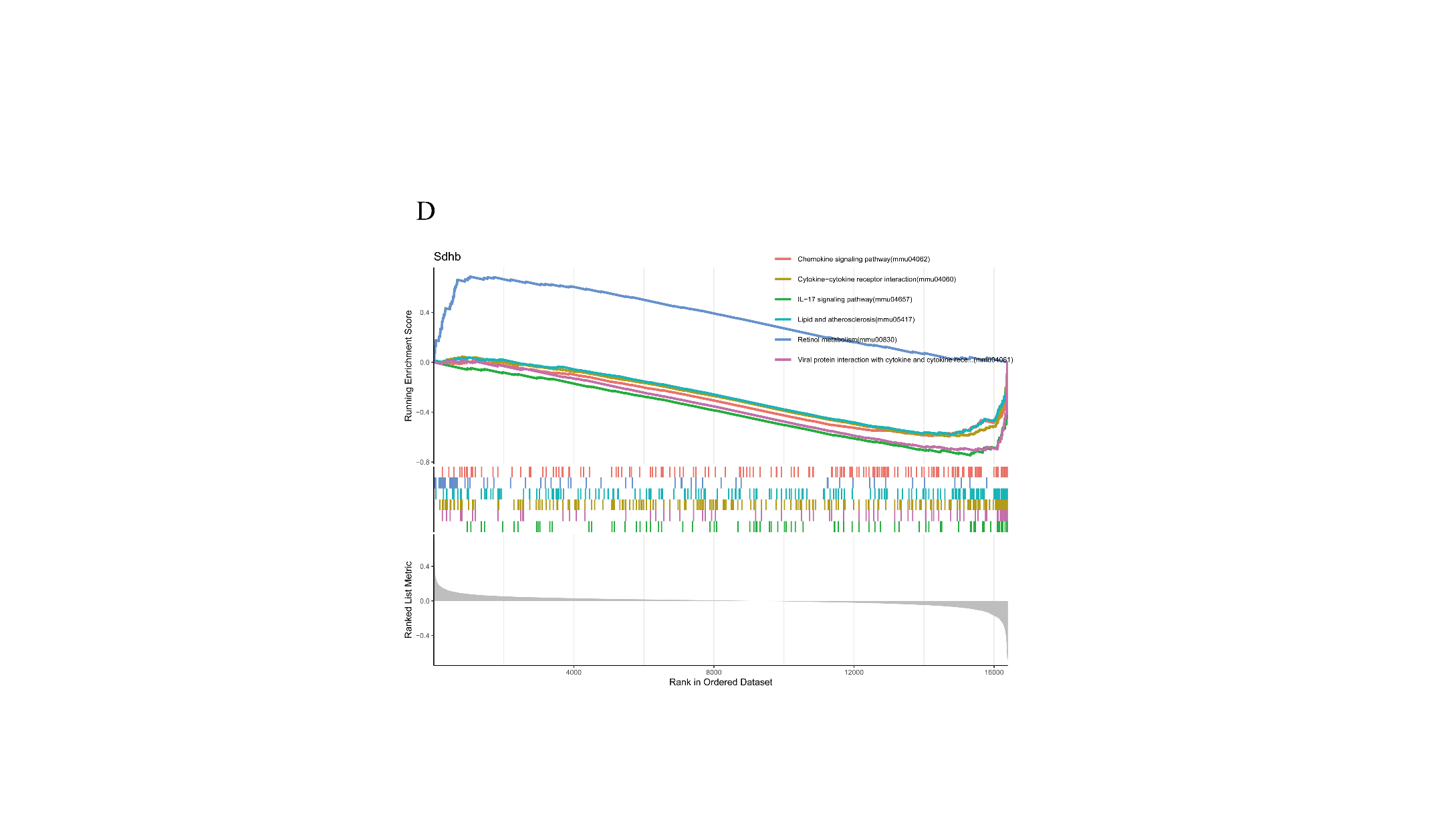


# Supplementary Figure 5

GSVA and GSEA of the OFGs based on the GO enrichment. GO enrichment analysis of (A) NDUFB2, (B) NDUFB6, (C) PDHA1, and (D) SDHB was conducted using the GSVA method, and the top 50 are visualized according to the enrichment score. GO enrichment analysis of (E) NDUFB2, (F) NDUFB6, (G) PDHA1, and (H) SDHB was conducted using the GSEA method. GSVA, Gene set variation analysis; GSEA, gene set enrichment analysis; OFGs, optimal feature genes; GO, Gene Ontology.

5-A (NDUFB2)


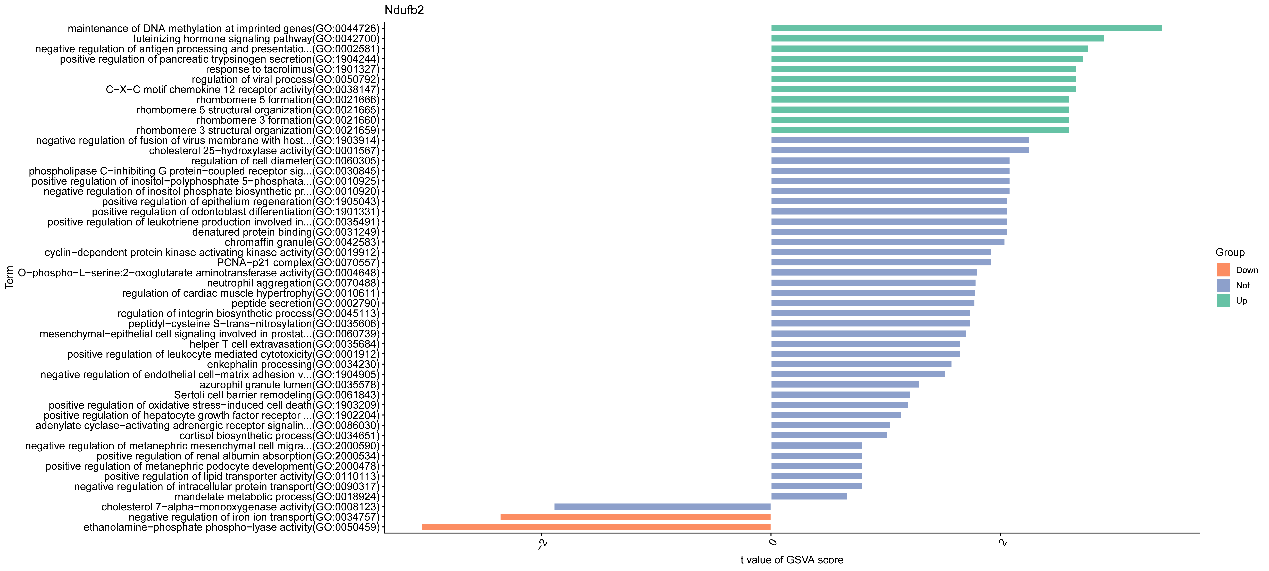


5-B (NDUFB6)


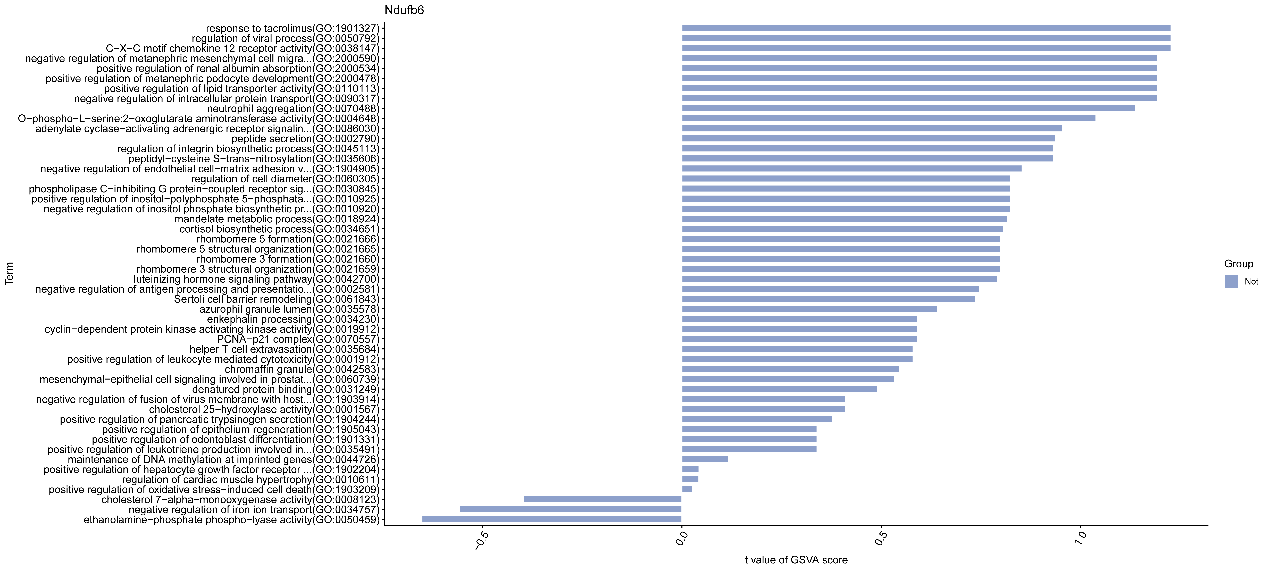


5-C (PDHA1)


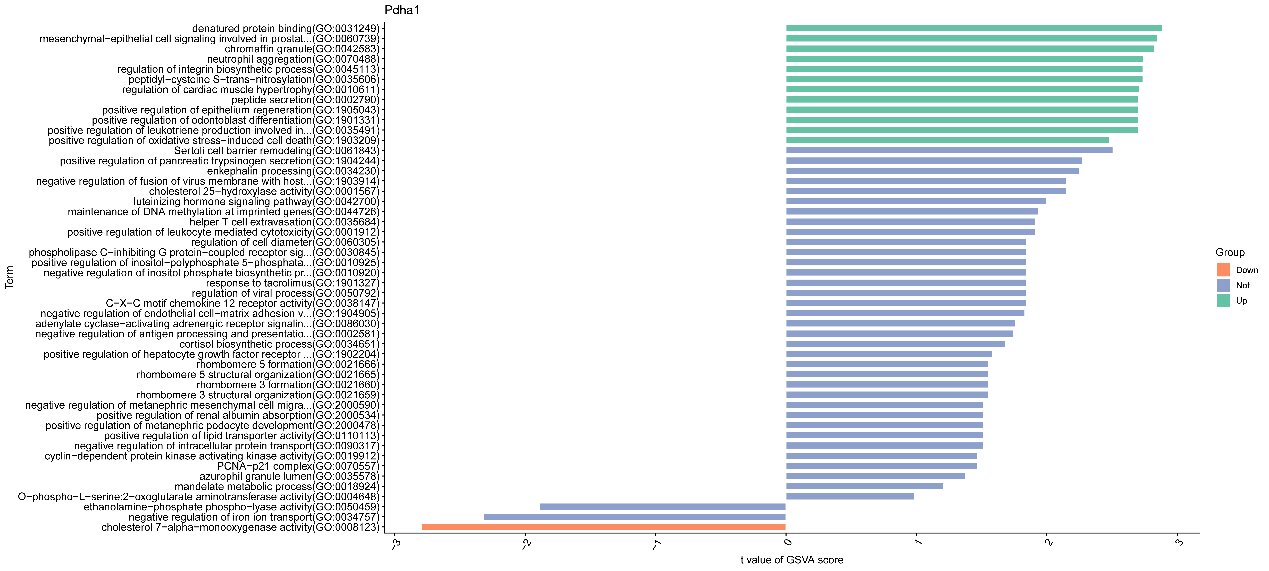


5-D (SDHB)


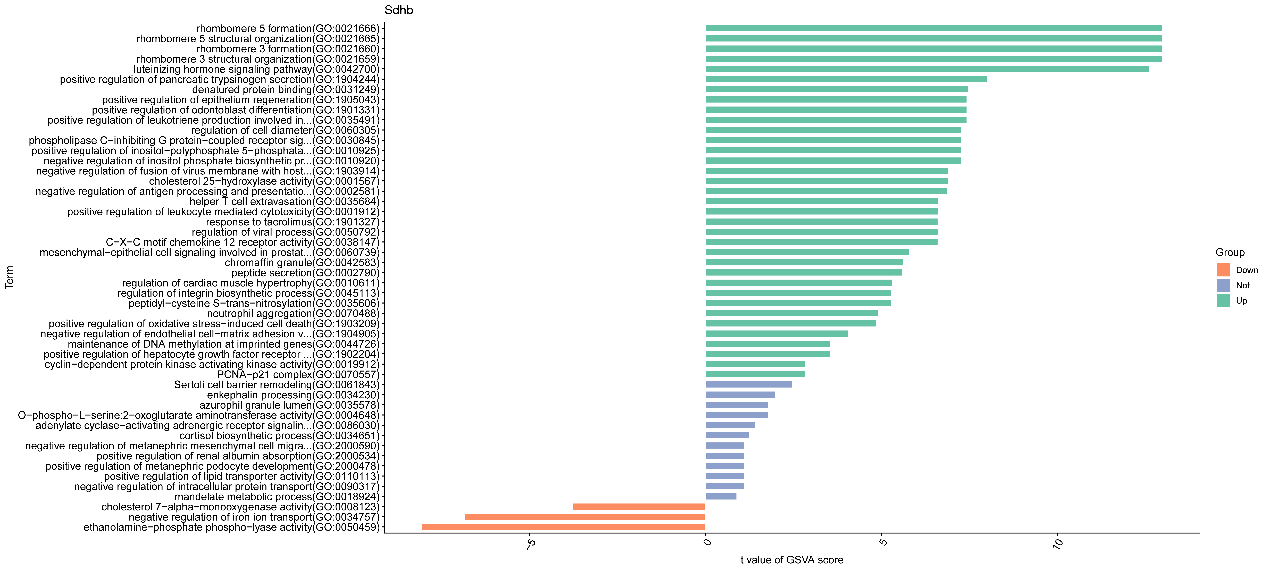


5-E (NDUFB2)


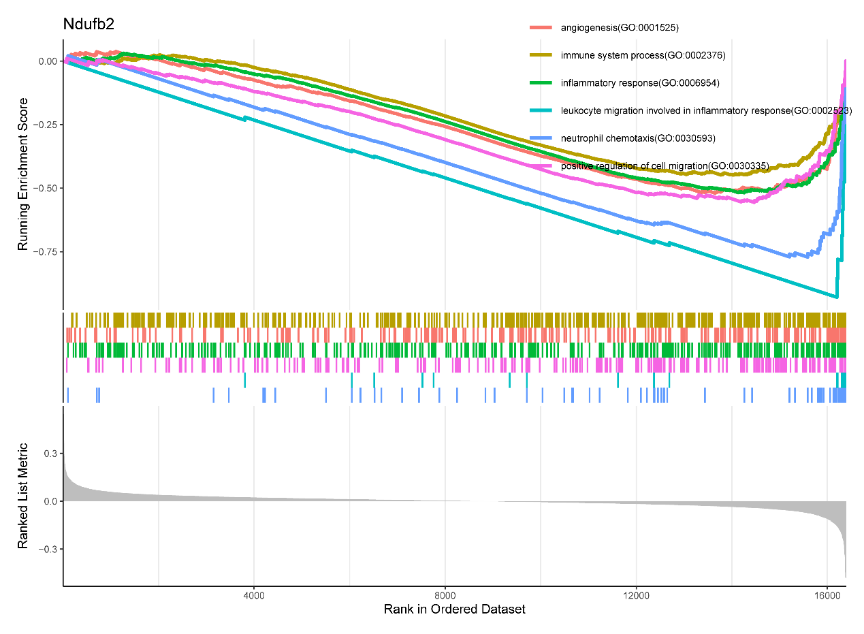


5-F (NDUFB6)


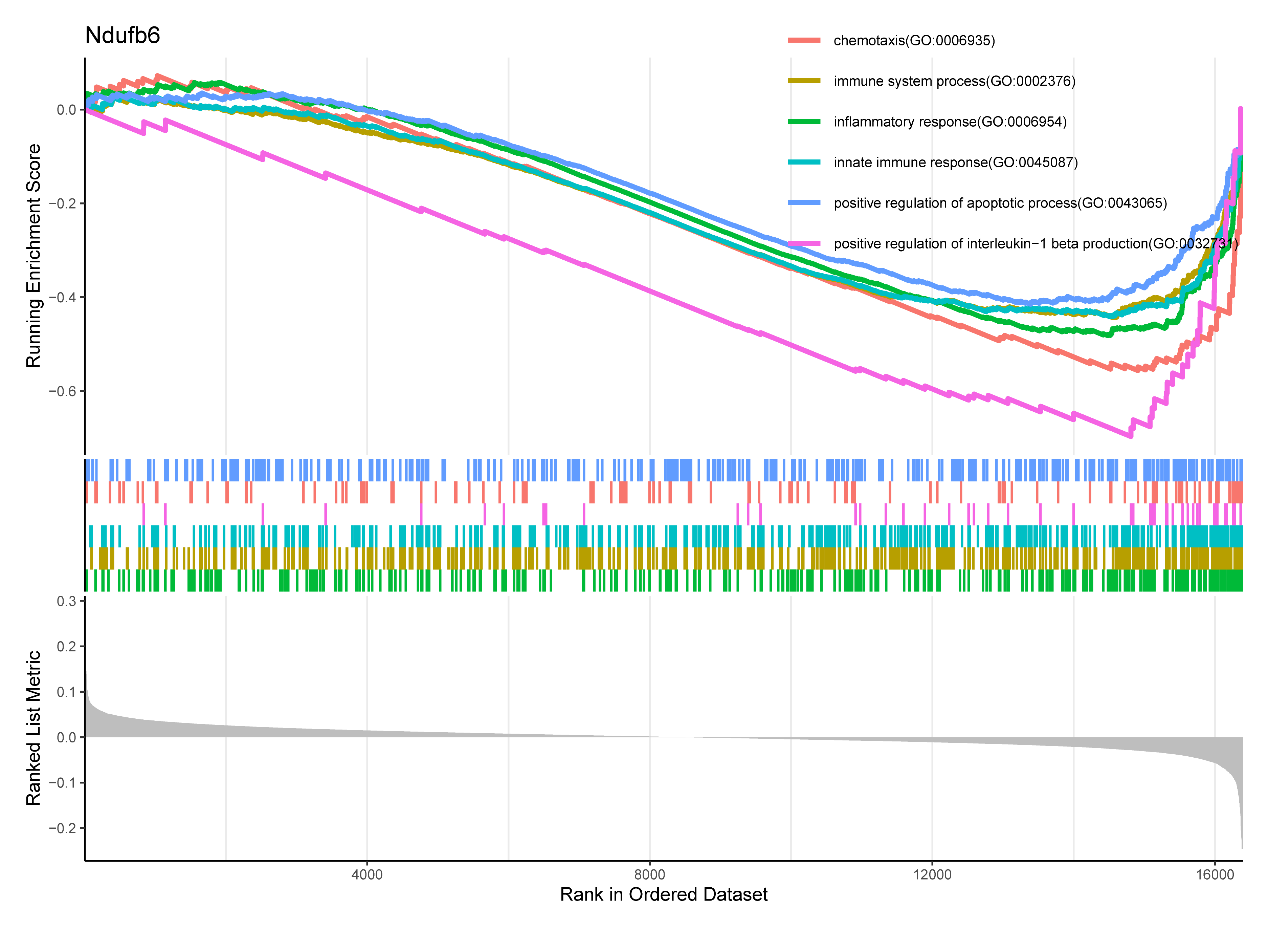


5-G (PDHA1)


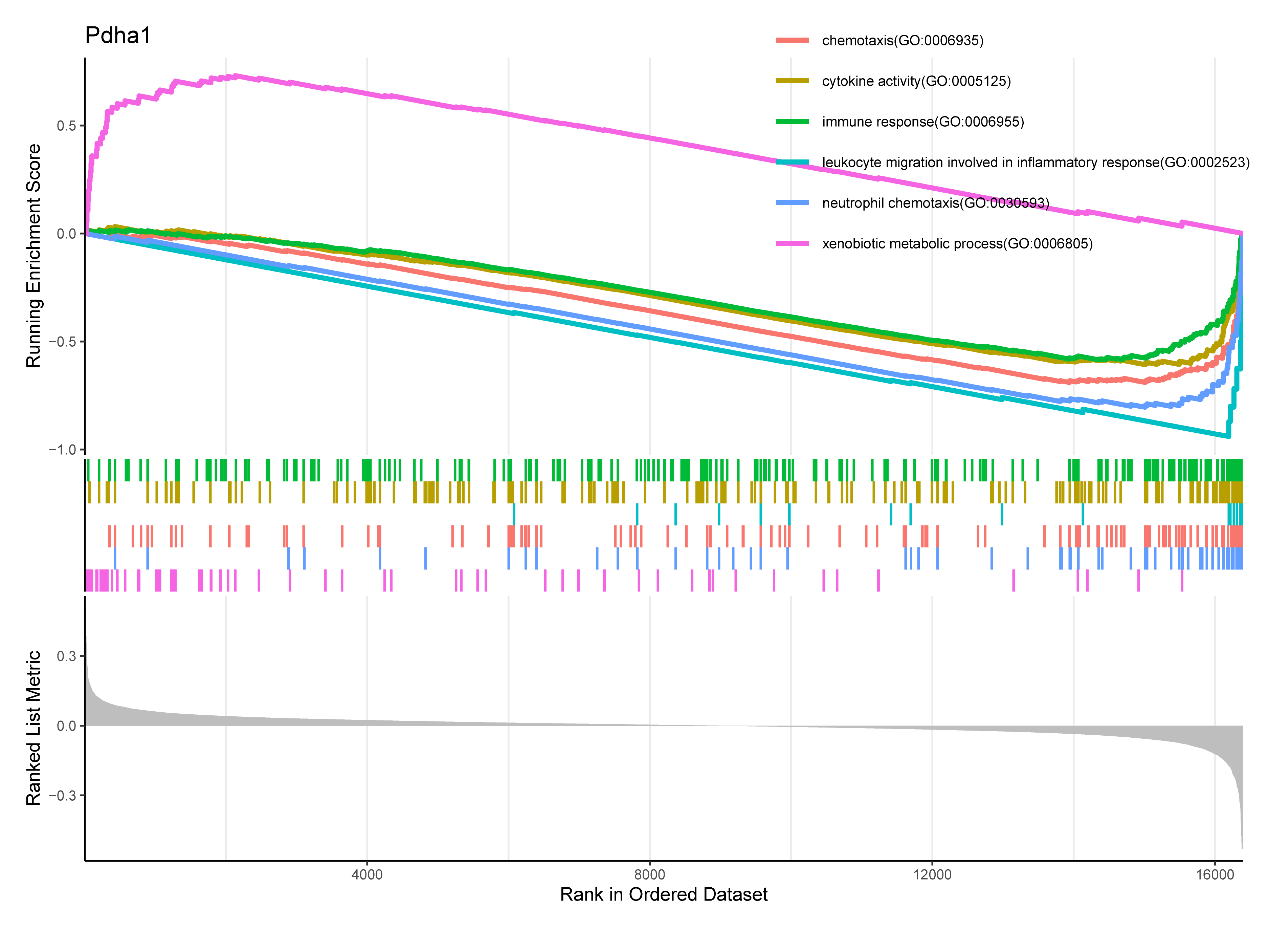


5-H (SDHB)


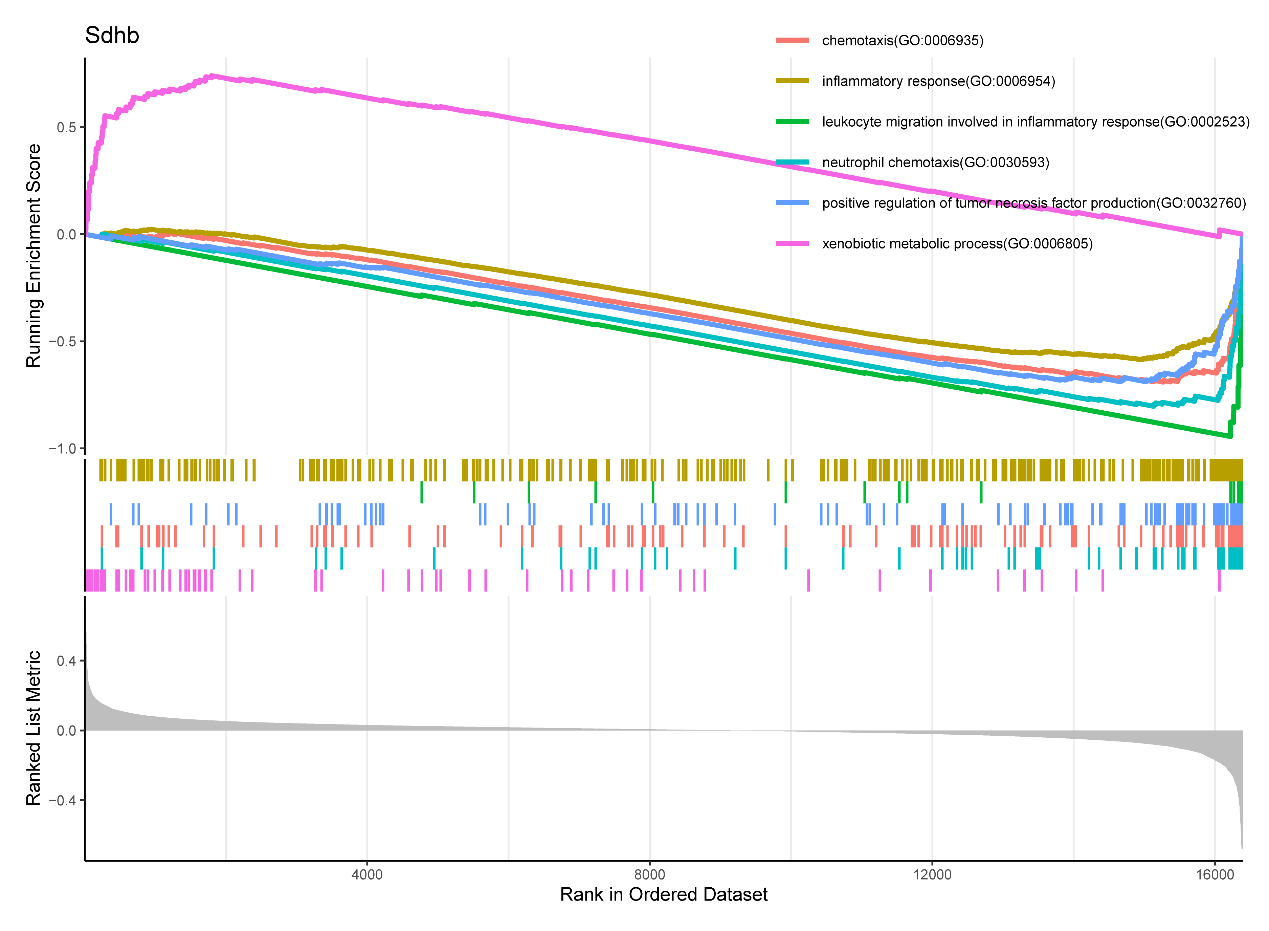


**Supplementary Figure 6**

Effects of ATTM on AILI. (A, B) Serum concentrations of ALT and AST (n = 6 per group). The results are expressed as the mean ± SD. ns, not significant. ALT, alanine aminotransferase; AST, aspartate aminotransferase; ATTM, ammonium tetrathiomolybdate.


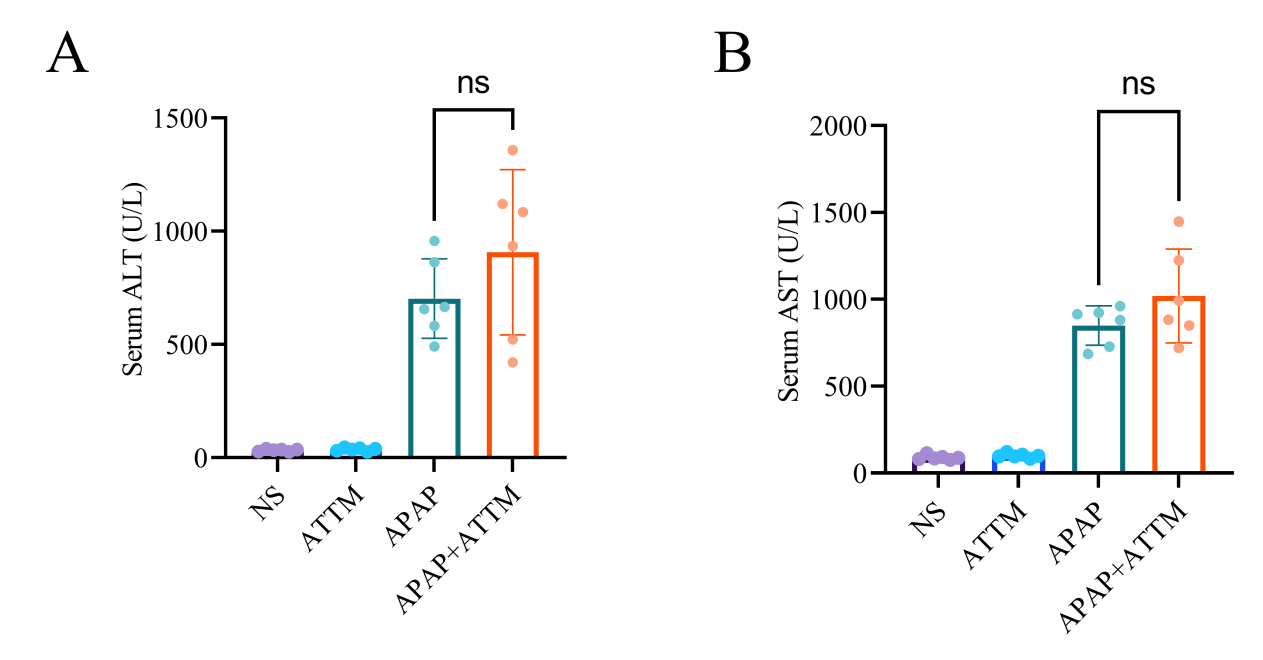

Supplement: Supplementary file 1 [file DataSheet_1.docx]
